# Ex Vivo Functional Characterization of Mouse Olfactory Bulb Projection Neurons Reveals a Heterogeneous Continuum

**DOI:** 10.1523/ENEURO.0407-24.2025

**Published:** 2025-02-28

**Authors:** Sana Gadiwalla, Chloé Guillaume, Li Huang, Samuel J. B. White, Nihal Basha, Pétur Henry Petersen, Elisa Galliano

**Affiliations:** ^1^Department of Physiology, Development and Neuroscience, University of Cambridge, Cambridge CB23EL, United Kingdom; ^2^Department of Anatomy, Biomedical Center, Faculty of Medicine, University of Iceland, Reykjavik 102, Iceland

**Keywords:** axon initial segment, excitability, mitral cells, olfactory bulb, parallel processing, tufted cells

## Abstract

Mitral cells (MCs) and tufted cells (TCs) in the olfactory bulb (OB) act as an input convergence hub and transmit information to higher olfactory areas. Since first characterized, they have been classed as distinct projection neurons based on size and location: laminarly arranged MCs with a diameter larger than 20 µm in the mitral layer (ML) and smaller TCs spread across both the ML and external plexiform layers (EPL). Recent in vivo work has shown that these neurons encode complementary olfactory information, akin to parallel channels in other sensory systems. Yet, many ex vivo studies still collapse them into a single class, mitral/tufted, when describing their physiological properties and impact on circuit function. Using immunohistochemistry and whole-cell patch–clamp electrophysiology in fixed or acute slices from adult mice, we attempted to align in vivo and ex vivo data and test a soma size-based classifier of bulbar projection neurons using passive and intrinsic firing properties. We found that there is no clear separation between cell types based on passive or active properties. Rather, there is a heterogeneous continuum with three loosely clustered subgroups: TCs in the EPL, and putative tufted or putative MCs in the ML. These findings illustrate the large functional heterogeneity present within the OB projection neurons and complement existing literature highlighting how heterogeneity in sensory systems is preponderant and possibly used in the OB to decode complex olfactory information.

## Significance Statement

Mitral cells (MCs) and tufted cells (TCs) in the olfactory bulb (OB) have traditionally been either grouped due to their shared role in early odor processing or separated into distinct groups based on in vivo physiology and circuit connectivity. However, our ex vivo study in postweaning mice reveals a more complex picture. Rather than being clearly distinct or identical, MCs and TCs form a diverse continuum of morphological and functional properties. This variability may enable efficient processing of the wide range of odors animals encounter. These findings highlight the importance of considering nuanced differences when classifying neurons in the OB and more broadly in the brain.

## Introduction

To guide behavior, concurrent and complex information from the environment must be efficiently decoded and processed by parallel pathways. First described for nociception and vision, parallel processing is now recognized as a hallmark strategy of the brain across sensory systems (Gasser and Erlanger, 1929; Hubel and Wiesel, 1959; Nassi and Callaway, 2009). In the mammalian olfactory system, parallel processing is implemented via two classes of output neurons, mitral cells (MCs) and tufted cells (TCs), which bring odor information transduced in the olfactory epithelium and preprocessed in the olfactory bulb (OB) to higher olfactory areas. While often lumped together and collectively referred to as mitral/tufted cells (M/TCs), recent work has started to highlight that these two classes of projection neurons are rather different.

MCs are laminarly arranged in the mitral layer (ML) and are known to have the largest somatic diameter (>20 µm) in the main OB (Nagayama et al., 2014; Imamura et al., 2020). Conversely, TCs are diffusely located throughout the external plexiform layer (EPL). TCs are further subdivided into groups based on the their soma position: superficial, middle, internal/deep, and from closest to furthest from the glomerular layer (GL), respectively (Schneider and Macrides, 1978; Orona et al., 1984; Nagayama et al., 2014). It should be noted that internal TCs are sometimes called “displaced MCs” due to their proximity to the ML (Mori et al., 1983; Ma et al., 2013). The TC soma diameter ranges between 10 and 20 µm and is not correlated to their EPL location (Pinching and Powell, 1971; Fukunaga et al., 2012; Nagayama et al., 2014).

Beyond the soma size, MCs and TCs are very similar morphologically, both having a primary apical dendrite which ends with a tuft into a single glomerulus (Pinching and Powell, 1971). However, they are differentially connected within the OB network, and superficial TCs receive stronger excitatory inputs from olfactory sensory neurons (OSNs) than MCs (Jones et al., 2020), as well as inhibitory drive from interneurons in the glomerular and granular layers (Christie et al., 2001; Geramita et al., 2016; Liu et al., 2019). Additionally, in higher olfactory areas, MCs reach wider territories of the piriform cortex (Nagayama, 2010; Igarashi et al., 2012), a difference recapitulated at the molecular level by a differential expression of axon guidance genes (Zeppilli et al., 2021).

Functionally, in vivo studies have shown that MCs and TCs encode complementary information thanks to different biophysical characteristics (Balu et al., 2004; Nagayama et al., 2004; Padmanabhan and Urban, 2010; Angelo and Margrie, 2011; Burton and Urban, 2014; Cavarretta et al., 2018; Ackels et al., 2020). Specifically, MCs encode odor concentration, while TCs play a key role in odor discrimination (Fukunaga et al., 2012; Igarashi et al., 2012; Burton and Urban, 2014; Nagayama et al., 2014, but see Chae et al., 2022). As such, TCs exhibit greater firing rates than MCs and have a shorter latency for odor response (Nagayama et al., 2004; Igarashi et al., 2012; Ackels et al., 2020).

Yet, despite this substantial in vivo evidence of diversity, few ex vivo studies discriminate between MCs and TCs and instead collapse them into an M/TC group. This lack of specificity between OB projection neurons, which is somewhat unsurprising given that the generation of specific transgenic mouse lines is very recent (Koldaeva et al., 2021), makes it difficult to align results across studies. A decade ago, Burton and Urban started to fill this gap by showing how internal TCs substantially differ from very large neurons in the ML (putative MCs, pMCs) in preweaning mice (Burton and Urban, 2014). This seminal study left three important questions unanswered: (1) whether the 20-µm-diameter classifier often used maps onto their data, (2) whether smaller cells in the ML are more similar to EPL TCs or to MCs, and (3) whether their results persist in adult animals given that in the first postnatal weeks, both TCs and MCs are still undergoing developmental changes (Yu et al., 2015; Tufo et al., 2022). To answer these questions and to harmonize recent in vivo studies with ex vivo work performed in mouse lines without a specific fluorescent tag for MCs or TCs, this study aims to uncover whether the 20-µm-diameter classifier coupled with the intrinsic physiological features of OB projection neurons is sufficient to unequivocally classify MCs and TCs across all layers.

## Materials and Methods

### Animals

Mice of either sex are housed under a 12 h light/dark cycle in an environmentally controlled room with *ad libitum* access to food and water. In line with the 3R principles, we used both wild-type mice (C57Bl/6J; Charles River Laboratories) and surplus animals from transgenic breedings [DAT^IREScre^ (B6.SJL-Slc6a3^tm1.1(cre)Bkmn^/J, Jax stock #006660] and Ai9 [B6.Cg Gt(ROSA)26Sor^tm9(CAG-tdTomato)Hze/^J; Jax stock #007909)] ongoing in the laboratory. Similarly, while almost all mice were postweaning juveniles aged between Postnatal Day (P)20 and P40, for electrophysiology experiments, we also included 7 cells out of a total of 92 (7.6% total, of which 6pMC and 1 eplTC) from mice aged P43, P44, P53, and P68, after confirming the lack of properties of clustering based on age. The integrity of the transgenic lines was ensured by generating breeders via back-crossing heterozygous carriers with C57Bl6 animals specifically bought biannually from Charles Rivers Laboratories. All experiments were performed at the University of Cambridge in accordance with the Animals (Scientific procedures) Act 1986 and with AWERB (Animal Welfare and Ethical Review Board) approval.

### Immunohistochemistry

Mice were anesthetized with a lethal dose of pentobarbital and perfused with 20 ml PBS with heparin (20 ml units.ml^−1^), followed by 20 ml of 1% paraformaldehyde (PFA; in 3% sucrose, 60 mM PIPES, 25 mM HEPES, 5 mM EGTA, and 1 mM MgCl_2_). OBs were dissected and postfixed in 1% PFA for 2–7 d and embedded in 5% agarose and sectioned into 50 µm slices using a vibratome (VT1000S, Leica Biosystems). Free-floating slices were washed with PBS and incubated in 5% normal goat serum in PBS/Triton X-100/azide (0.25% Triton X-100, 0.02% azide) for 2 h at room temperature and then incubated in primary antibody solution (in PBS/Triton X-100/azide) for 2 d at 4°C. Primary antibodies and their working dilutions included SMI-32 neurofilament H nonphosphorylated (SMI-32; mouse, BioLegend; 1:1,000), ankyrin-G (AnkG; guinea pig, Synaptic Systems, 1:500), and tyrosine hydroxylase (TH; rabbit, Sigma Millipore AB152, 1:500). Following primary incubation, slices were washed three times in PBS for 5 min before secondary antibody solution (species-appropriate, Life Technologies, Alexa Fluor-conjugated) 1:1,000 in PBS/Triton X-100/azide for 3 h at room temperature. Slices were then washed in PBS and incubated in 0.2% Sudan black in 70% ethanol at room temperature for 3 min to minimize autofluorescence and mounted on glass slides (Menzel-Gläser) with FluorSave (Merck Millipore).

### Fixed tissue imaging and analysis

**Figure 1.**
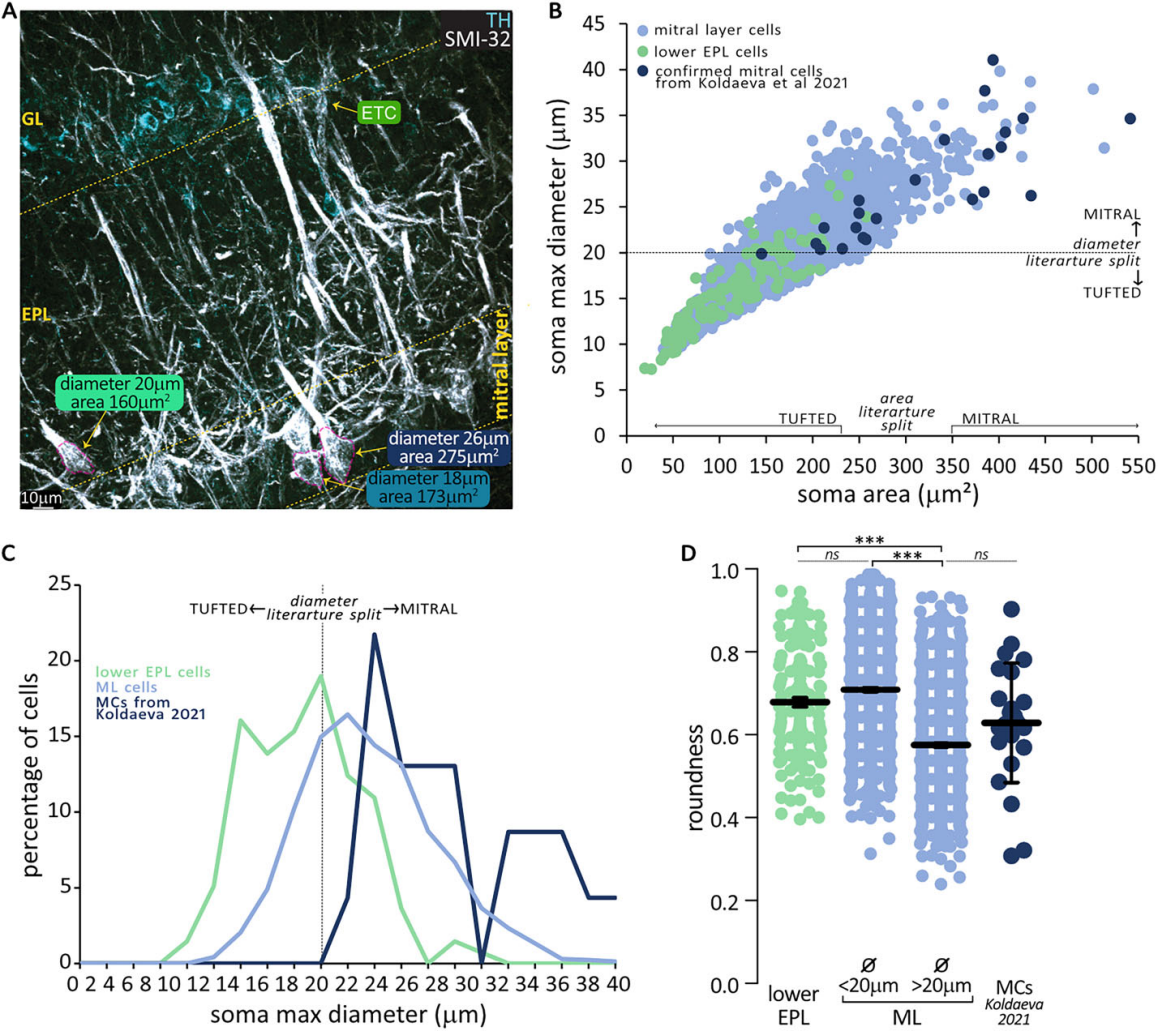
OB projection neurons in the ML include differently sized and shaped cells. ***A***, An example image of the OB outer layers. Excitatory projection neurons labeled with antibodies against neurofilament marker protein (SMI-32, white) span the EPL and the ML. Dopaminergic interneurons stained with antibodies against TH (cyan) indicate the location of the GL, adjacent to which are the SMI-32 positive somas of the excitatory interneurons external TCs (ETCs). The soma of three representative projection neurons across EPL and ML has been manually traced in dashed magenta to calculate the maximum diameter and area. ***B***, Correlation of the soma area and maximum diameter for OB projection neurons located in the lower EPL (green, *n* = 137) or ML (cyan, *n* = 1,671). Blue circles represent confirmed MCs from the Lbhd2-CreERT2 transgenic mouse line, meta-analyzed from Koldaeva et al. 2021 (*n* = 23). The canonical diameter and area dividers between MCs and TCs are indicated on the axes. ***C***, Frequency distribution of maximum diameters for OB projection neurons located in the lower EPL (green, *n* = 137), ML (cyan, *n* = 1,671), and confirmed MCs from the Lbhd2-CreERT2 transgenic mouse line. ***D***, Soma roundness [(4 * area / (π * major_axis^2^)] of EPL TCs (green) and confirmed MCs (blue) compared with ML cells (cyan) when split by the 20 µm max diameter classifier proposed in the literature. Circles are individual cells; lines are mean ± SEM; ***p < 0.001; *ns*, not significant.

Images were acquired with a laser scanning confocal microscope (Carl Zeiss LSM 900) using appropriate excitation and emission filters, a pinhole of 1 AU, and a 40× oil immersion objective. Laser power and gain were set to prevent signal saturation in channel images for localization analysis. All quantitative analysis was performed with Fiji (ImageJ). We identified M/TCs with antibodies against the neurofilament marker protein, SMI-32, which labels the cell body, axon, and dendrites of bulbar excitatory neurons. To unequivocally define the upper border of the EPL, we costained the tissue with antibodies against TH to label GL's dopaminergic neurons (Fig. 1*A*). For soma size analysis, images were taken with a 1× zoom (0.415 µm/pixel) and 512 × 512 pixels in *z*-stacks with 1 µm steps. In all animals, images were sampled from the rostral third, middle third, and caudal third of the OB. To avoid selection bias, all cells present in the stack and positive for the SMI-32 were measured using Fiji/ImageJ. Soma area, Feret's diameter (i.e*.*, the longest distance between any two points), roundness (4 * area / (π * major axis^2^), and circularity (4π * area / perimeter^2^) were measured at the single plane including the cell's maximum diameter by drawing an ROI with the freehand drawing tool. Cells were classified based on their location within the ML and EPL. If their soma fell clearly within the boundaries of the ML, it was determined to be a putative mitral or TC. Alternatively, neurons whose soma was fully located in lower third of the EPL (i.e., the EPL third closest to the ML) were classed as EPL TCs.

For axon initial segment identification, images were taken with 2× zoom, 512 × 512 pixels (0.138 µm/pixel) in *z*-stacks with 0.30 µm steps. Laser power and gain settings were adjusted to prevent signal saturation in the axon initial segment label AnkG. The cellular marker SMI-32 signal which labels tufted excitatory neurons (Hamada et al., 2016; Galliano et al., 2021) was usually saturated to enable clear delineation of the axon. Distance from soma and length was measured in Fiji/ImageJ using the View5D plugin which allows for 3D manual tracing of cell processes. The axon initial segment distance from soma was calculated as the neurite path distance between its start (proximal part where AnkG staining was clearly identifiable) and the start of its primary parent process (the axon). Axon initial segment length was calculated by following AnkG staining along the course of the axon from the AIS start position to the point where AnkG staining was no longer identifiable and only SMI-32 straining was present along the axon.

### Electrophysiology

Mice were decapitated under isoflurane anesthesia. The brain was removed and transferred into ice-cold slicing medium containing the following (in mM): 240 sucrose, 5 KCl, 1.25 NaH_2_PO_4_, 2 MgSO_4_, 1 CaCl_2_, 26 NaHCO_3_, and 10 D-glucose, bubbled with 95% O_2_ and 5% CO_2_. Horizontal slices (300 µm) of the OB were cut using a vibratome (Campden Instruments 7000SMZ-2 or Leica VT1000S) and stored in ACSF containing the following (in mM): 124 NaCl, 2.5 KCl, 1.25 NaH_2_PO_4_, 2 MgSO_4_, 2 CaCl_2_, 26 NaHCO_3_, and 15 D-glucose, bubbled with 95% O_2_ and 5% CO_2_ for at least 1 h at room temperature before experiments began.

Cells were visualized with an upright microscope (BX51W1; Olympus) using a 40× water immersion objective with a visible light camera (quantalux sCMOS; Thorlabs) and classified based on their location in the MCL and EPL. Cells were targeted for patch clamp if the entirety or over half of their soma lay between the boundaries of the MCL and had a max diameter >10 µm, i.e., bigger than that of the rare 5T4-expressing MCL granule cells (Batista-Brito et al., 2008). Internal TCs were targeted based on their location in the lower third of the EPL (Mori et al., 1983; Kishi et al., 1984; Shepherd, 2004).

Whole-cell patch–clamp recordings were amplified and digitized using an EPC-9 (HEKA Elektronik) or MultiClamp 700B and Digidata 1550B (Molecular Devices) at physiologically relevant temperatures (30 ± 2°C), maintained with an in-line heater (SH-27B and TC-344C, Warner Instruments). All signals were Bessel filtered at 10 kHz, with experiments recorded at 20 kHz and single-spike recordings filtered at 200 kHz. Recordings were excluded if series resistance (evaluated by −10 mV voltage steps following each test pulse) was <40 MΩ for putative MCs with a capacitance >40 pF and <30 MΩ for putative TCs with a capacitance <40 pF. Protocols were also excluded if these values varied >20% over the course of the experiment and holding current values exceeded −300 pA. Fast capacitance was compensated in on-cell configuration. Cell capacitance was calculated by measuring the area under the curve of a transient capacitive current induced by a −10 mV step after subtracting the steady-state current induced by the voltage pulse. Recording electrodes (30-0094 and 30-0062, Harvard Apparatus) were pulled using a horizontal or vertical puller (P-87, Sutter Instruments; PC-100, Narishige) to achieve a tip resistance of 1.5–3.0 MΩ (larger tips for putative mitral, smaller for putative tufted and EPL tufted) when filled with a potassium-gluconate intracellular solution containing the following (in mM): 124 K-gluconate, 9 KCl, 10 KOH, 4 NaCl, 10 HEPES, 28.5 sucrose, 4 Na_2_ATP, 0.4 Na_3_GTP, pH 7.25–7.35 (290 MOsm), and Alexa Fluor 488 (Thermo Fisher Scientific, 1:150).

To precisely measure the soma size and ensure fluorophore diffusion throughout the entire soma and confirm that the dendrites were not severely cut, cells were patched for at least 10–15 min prior to capturing a snapshot of their Alexa Fluor 488-filled soma and proximal dendrite (field of view size, 100.90 × 179.43 µm) with LED excitation (LED1B; Thorlabs) using the appropriate excitation and emission filters (ET575/50 m; CAIRN Research UK). Quantitative analysis was performed in Fiji/ImageJ. Diameter and area were calculated by drawing a ROI using the freehand drawing tool. In a subset of recordings, biocytin (Sigma-Aldrich; 2%) was added to evaluate morphology. These slices were fixed with 1% PFA in PIPES overnight and then incubated with 1:1,000 streptavidin Alexa Fluor 488 conjugate in PBS/Triton X-100/azide for 2 h at room temperature to reveal the biocytin filling.

In a current-clamp mode, experiments were only evaluated if their voltage was maintained stably at −60 ± 3 mV. For action potential (AP) measurements, injections of 10 ms current steps of increasing amplitudes were applied until current threshold was met, and the cell fired an AP (*V*_max _> 0 mV). Repetitive firing properties were measured with injections of 500 ms current steps starting at 0 mV of increasing amplitudes (5–35) until the neuron passed its maximum firing frequency. Sag potentials were evoked by injecting a 500 ms current injection starting from −300 to −700 pA. Exported traces were analyzed using either ClampFit (pClamp 10, Molecular Devices) or custom-written scripts in MATLAB (MathWorks).

### Quantification of passive and active electrophysiological properties

Quantification of AP properties was calculated from the first AP evoked by the weakest suprathreshold input. Current threshold was defined as the minimum threshold needed to elicit the first AP. Voltage threshold was taken as the potential at which dV/dt first passed 10 V/s and the maximum of depolarization. The peak was the highest voltage an AP reached. AP amplitude was the difference between the voltage threshold and the AP peak. Spike width was measured at the midpoint between voltage threshold and maximum voltage. Afterhyperpolarization (AHP) values were measured from responses to 500 ms current injection from the local voltage minimum after the first spike fired at rheobase.

For repetitive firing properties, input–output curves were created by counting the number of spikes fired at each level of injected current density. The slope of the input–output curve was measured between the first sweep with a non-zero AP and the sweep where the maximum number of APs was fired. The following parameters were measured only from the sweep with the maximum number of APs were fired: (1) first AP delay which measured the time interval between the start of the current injection and the peak of the first AP; (2) peak of the first AP; and (3) AP frequency. To measure the variability in the firing pattern, we calculated the coefficient of variance (CV) of the interspike interval (ISI) across current injections and at the sweep that fired the maximum number of APs. CV was calculated as the ratio of the standard deviation of ISIs to the mean ISI of the cell. Firing pattern variability measure CV2 was calculated as mean value of (2 * abs[(ISI_n _+ 1 − ISI_n_)] / (ISI_n _+ 1 + ISI_n_); Holt et al., 1996). To further analyze the temporal coding further, we binned the current injection into five 100 ms epochs, and the AP firing was evaluated along them (Goldfarb et al., 2007).

Sag potentials were evaluated as done previously (Angelo and Margrie, 2011) where sag index was calculated as the ratio between the peak (minimum within the first 100 ms) and steady-state (mean of final 50 ms) currents normalized to the holding voltage.

### Statistical analysis and data availability

Statistical analyses were carried out using Prism (GraphPad) or MATLAB (MathWorks). “*N*” refers to the number of animals, and “*n*” indicates the number of cells. Normality of sample distribution was assessed with the D'Agostino and Pearson’s omnibus test and their parametric or nonparametric tests used accordingly. All comparisons were two-tailed. Multiple comparisons were performed among all groups, and post hoc tests after nested ANOVAs or Kruskal–Wallis were done using Tukey's/Dunn's or the two-stage linear step-up false discovery rate (FDR) procedure of Benjamini, Krieger, and Yekutieli. The α values were set at 0.05, and on the figures only significant differences are indicated with star(s). K-means clustering of immunohistochemistry and electrophysiology data were executed in MATLAB (MathWorks) using custom-written scripts which included an evaluation of silhouette coefficient. Cluster numbers were unbiased, chosen based on the silhouette coefficient closest to one. Principal component analysis (PCA) of electrophysiological data was performed in Prism (GraphPad) for all cells with recordings passing inclusion criteria for all three of these protocols: (1) passive properties, (2) single AP properties, and (3) repetitive firing. Principal components (PCs) were selected based on the Kaiser rule, where PCs were selected only if they had an eigenvalue greater than one. All PCAs were unsupervised. Loading scores were calculated based on standardized data using the following formula: [Eigenvector * sqrt(Eigenvalue)]. Each cell was colored on the plot, post hoc allowing for a visual assessment of functional clusters. Postpublication, the full dataset will be released on the University of Cambridge Apollo repository (https://doi.org/10.17863/CAM.114886) under a CC-BY license.

## Results

### Soma size and morphology of mitral and TCs is heterogeneous

Two criteria have been traditionally used to classify MCs and TCs: the location and size of their somas. There is historical consensus in the literature that MC somas lie entirely or predominantly in the ML and that they are large, with a longest-axis diameter >20 µm and a corresponding area >350 µm^2^. Conversely, TC somas are spread across the EPL and on average smaller than MCs—longest-axis diameter <20 µm, area <230 µm^2^ (Mori et al., 1983; Orona et al., 1984; Ezeh et al., 1993; Royet et al., 1998; Nakajima et al., 2001; Shepherd, 2004; Nagai et al., 2005; Fukunaga et al., 2012; Igarashi et al., 2012; Nagayama et al., 2014).

We first investigated whether this historical diameter boundary could be used as a reliable binary classifier of mitral versus tufted identity in the fixed OB tissue. Using confocal microscopy, we acquired 3D *z*-stacks where we sampled SMI-32-positive neurons in the lower third of the EPL (i.e., internal TCs) and in the ML. We found that TCs in the EPL had a mean diameter of 15.73 µm and a mean area of 115.4 µm^2^ (Fig. 1*B*, green circles). In the ML, however, we found that soma sizes were more heterogeneous—mean diameter, 20.61 µm; range, 9.46–39.79 µm; mean area, 180.4 µm^2^; range 19.47–513.22 µm^2^ (Fig. 1*B*, cyan circles; area-diameter linear regression *R*^2 ^= 0.74; soma diameter frequency distribution; Fig. 1*C*). To further describe this heterogeneity and given that MCs have been further divided according to soma shape (Kikuta et al., 2013), we calculated the soma roundness at its longest-axis diameter. Smaller cells in the ML (diameter <20 µm) were rounder than ML cells with a diameter >20 µm, but they did not differ in roundness nor in the area from EPL TCs (Kruskal–Wallis with Dunn's corrections; *p* < 0.0001; Fig. 1*D*). Moreover, when we performed a secondary analysis of published data in confirmed MCs labeled in the Lbhd2-CreERT2 transgenic mouse line (Koldaeva et al., 2021; *n* = 23; mean diameter, 27.22 µm; mean area, 317.868 µm^2^; mean roundness ,0.63; Fig. 1*B–D*, dark blue dots/line), we found that these neurons were similarly sized and shaped as the largest ML cells in our dataset (one-way ANOVA nested on mouse or figure with Tukey's corrections; roundness *F_(_*_3,22)_ = 32.95; pMC vs Koldaeva et al., 2021 *p* = 0.32; area, *F_(_*_3,22)_ = 14.90; pMC vs Koldaeva et al., 2021 *p* = 0.05; diameter, *F_(_*_3,22)_ = 16.56; pMC vs Koldaeva et al., 2021 *p* = 0.64).

In summary, our data are broadly in agreement with the literature in that TCs in the EPL are round and have on average somas smaller than 20 µm and that the ML contains some very large and more ovoidal neurons. However, we found no clean split as advocated in previous studies: first, a minority of large soma neurons are present in the EPL, and second, over a third of ML neurons are small and round. These latter cells are more similar in size and shape to EPL TCs than ML MCs, and we tentatively class as putative ML TCs (pTCs).

### Unbiased k-means analysis identifies a capacitance threshold to classify mitral and TCs

**Figure 2.**
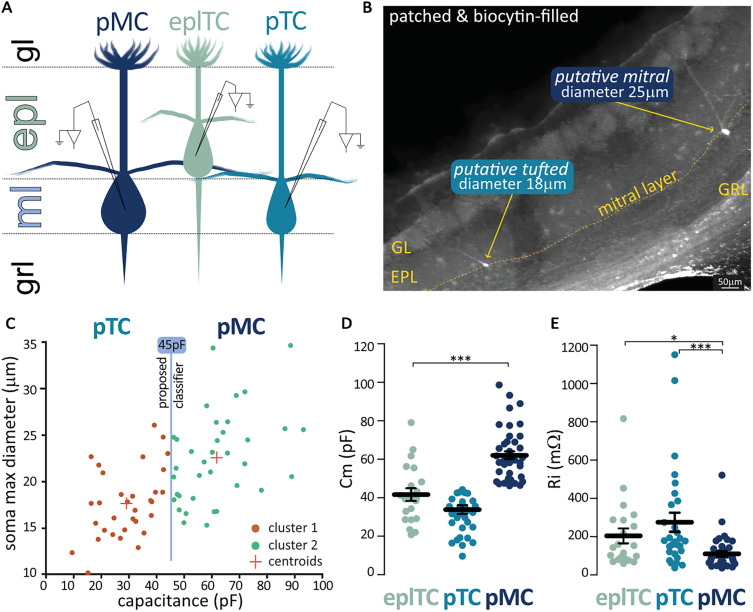
The soma size and passive electrical properties differ between OB projection neurons. ***A***, Schematic representation of the location of OB projection neurons targeted for whole-cell patch–clamp recordings in acute horizontal mouse brain slices. ***B***, Two cells in the ML, a putative tufted (pTC) and a putative mitral cell (pMC) patched with biocytin-supplemented intracellular solution and postfixed for morphological analysis. ***C***, Unbiased *k*-means analysis of the soma size of patched cells in the ML returns two clusters separable by a 45 pF capacitance classifier. ***D–E***, Membrane capacitance (Cm) and input resistance (Ri) in eplTC (*n* = 21) and ML's pTC (*n* = 28) and pMC (*n* = 42) classed using the 45 pF divider. Circles are individual cells; lines are mean ± SEM; *p < 0.05; ***p < 0.001. GL, glomerular layer; EPL, external plexiform layer; GRL, granule cell layer.

Are pTCs in the ML not only morphologically but also physiologically like EPL TCs? To evaluate their electrophysiological properties, we performed whole-cell patch clamp in acute slices, enabling precise determination of soma location (Fig. 2*A*) and subsequent measurement following intracellular labeling with fluorescent markers (Fig. 2*B*). Recognizing the well known disparity between live and fixed samples due to fixation-induced tissue shrinkage (Boonstra et al., 1983) and given that our earlier analysis failed to reveal a clear separation using the 20-µm-diameter classifier in the fixed tissue (Fig. 1*B*–*C*), we decided to adopt an alternative classification strategy. To correlate morphology with electrophysiology and ultimately distinguish between pMCs and pTCs based on soma size-related passive properties, we implemented an unbiased *k*-means algorithm based on capacitance, a reliable indicator of the somatic size. We input the diameter and capacitance of recorded ML cells into the *k*-means algorithm, which yielded two distinct clusters with centroids at 17.64 µm diameter/29.23 pF capacitance (representing pTCs) and 22.51 µm diameter/61.87 pF capacitance (representing pMCs). These two clusters could be separated by a capacitance-based classifier of 45 pF (Fig. 2*C*). Using this fully unbiased classifier, we compared mean capacitance of pTCs and pMCs with those of TCs recorded in the EPL (41.63 ± 3.32 pF; max diameter, 18.49 ± 0.89 µm) and confirmed that only pMCs are significantly different (Kruskal–Wallis test with Dunn's corrections eplTCs vs pMCs *p* < 0.001; Fig. 2*D*). In line with this result and in further support of the capacitance classifier, we found that input resistance (*R*_i_, also partially dependent on the soma size) was significantly lower in pMCs than in both pTCs and eplTCs (eplTC 205 ± 39 mΩ, pTC 275 ± 50 mΩ, pMC 110 ± 13 mΩ; Kruskal–Wallis test *p* < 0.001; Dunn's corrections eplTCs vs pMCs *p* < 0.05; pTCs vs pMCs *p* < 0.001; Fig. 2*E*).

In summary, we successfully identified a purely electrophysiological measure—capacitance—that can be used in the live tissue to attempt a classification of ML cells into the two subgroups.

### Sag voltages are not significantly different between principal neurons in EPL and ML

**Figure 3.**
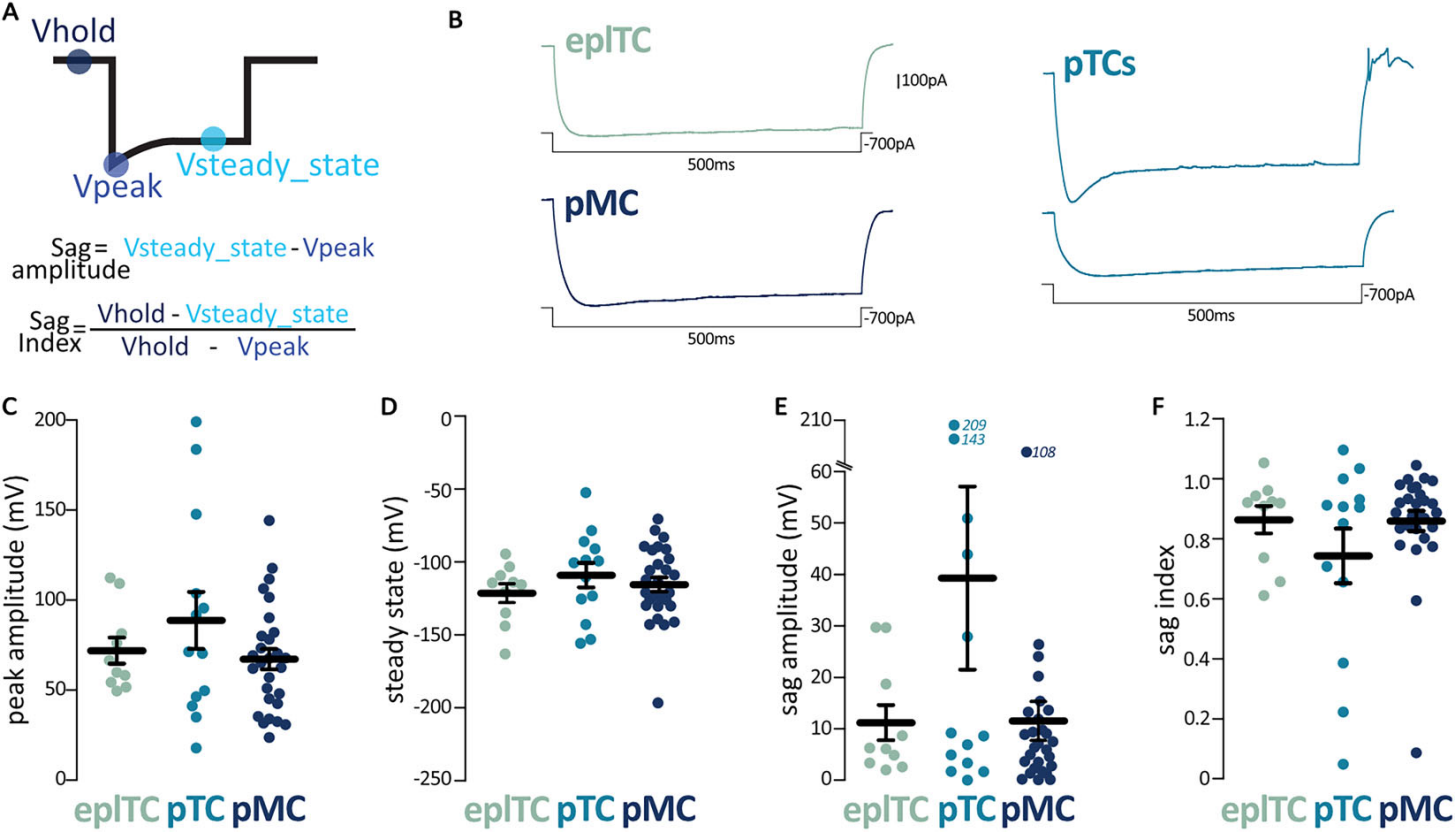
The depolarization response to hyperpolarization (sag potential) is extremely variable among OB projection neurons. ***A***, Schematic visualization of the voltage sag analysis parameters and formulas used to calculate sag amplitude and index. ***B***, Example traces of the voltage sag response to hyperpolarizing current injections in eplTC (green, *n* = 10), pTCs (cyan, *n* = 13), and pMC (blue, *n* = 28). Note the variability in pTCs. ***C–F***, Peak amplitude, steady-state voltage, sag amplitude, and sag index in the three classes of OB projection neurons. Circles are individual cells; lines are mean ± SEM; further quantification and statistical analysis in Table 1.

The presence of a hyperpolarization-activated cation current (*I*_h_/sag currents), which are important determinants of a neuron's intrinsic excitability (Combe and Gasparini, 2021), have been shown to be variable in ML cells (Angelo and Margrie, 2011; Angelo et al., 2012). To assess if this reported heterogeneity mapped on the pTCs/pMCs subclasses, we injected increasing levels of hyperpolarizing current into ML and EPL principal neurons (Fig. 3*A*,*B*). We found no differences between pMCs, pTCs, and eplTCs in the raw measurements of sag peak and steady-state voltage nor in the combined measure of sag amplitude and index (Angelo and Margrie, 2011; Fig. 3*C–F*; ANOVA with Tukey or Kruskal–Wallis with Dunn; all *p* > 0.2; Table 1). Of note is the variability across all groups but especially the pTCs (sag amplitude interquartile range and CV: eplTCs, 18.25 mV, −97%; pTCs, 4 4.881 mV, −163%; mTCs, 10.48 mV, −174%; sag index interquartile range and CV: eplTCs, 0.23 mV, −17%; pTCs, 0.44 mV, −44%; mTCs, 0.13 mV, −21%; Bartlett's test for equal variances, amplitude *p* < 0.001; index *p* < 0.01). Across all three groups, approximately half of the cells fire on rebound following the hyperpolarization-induced voltage sag (eplTCs, 50%; pTCs, 58%; pMCs, 48%; *χ*^2^ test, *p* = 0.33).

Overall, our data confirm the sag variability reported in the literature, but this heterogeneity does not map onto the pTC and pMC subgroups.

### Higher AP threshold and more distal axon initial segment in eplTCs than in ML neurons

**Figure 4.**
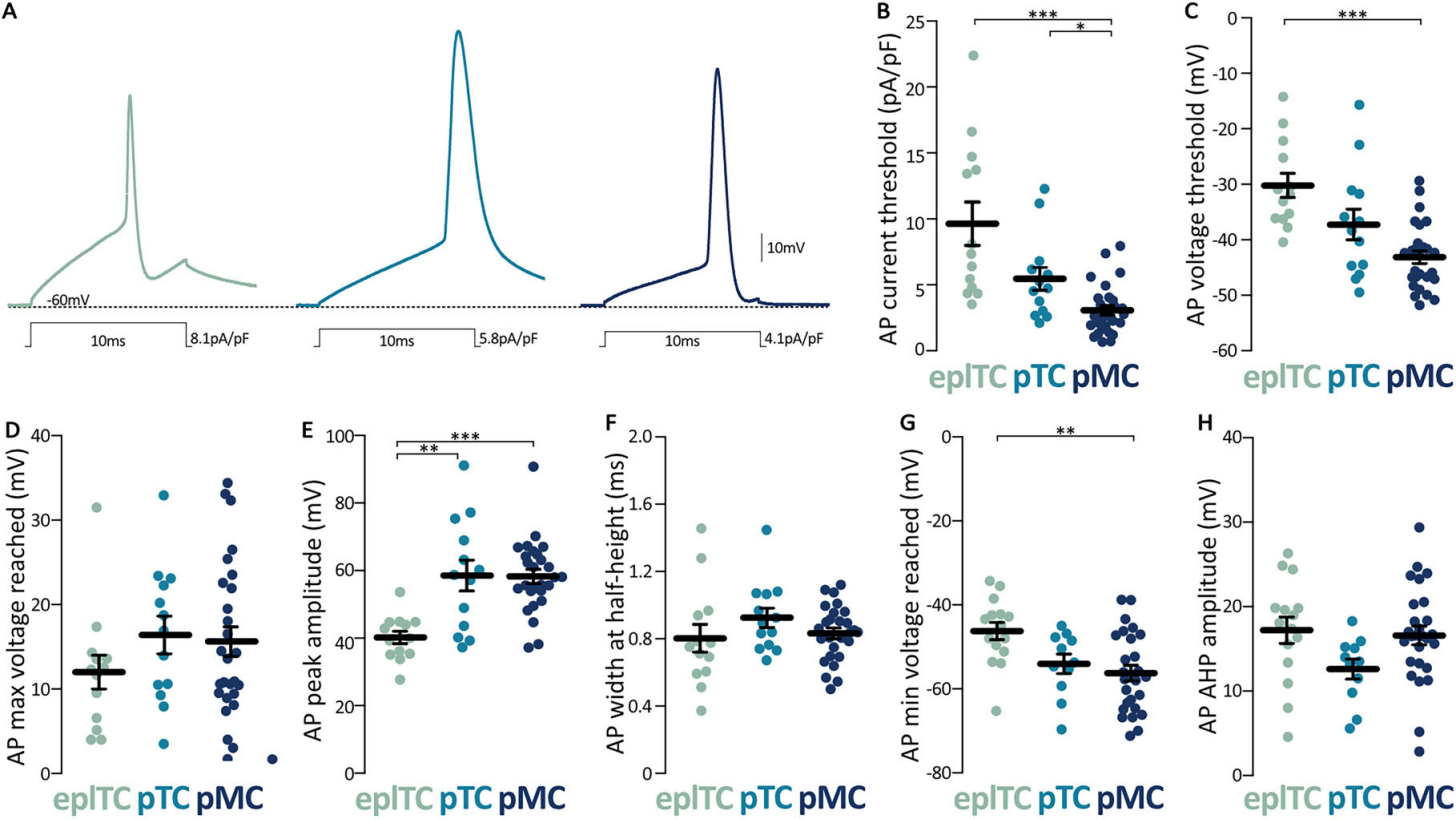
AP threshold and waveforms differ between OB projection neurons. ***A***, Example traces of the membrane voltage response to the minimum depolarizing 10-ms-long current injection needed to evoke an AP in eplTCs (green, *n* = 13), pTCs (cyan, *n* = 13), and pMCs (blue, *n* = 27). Waveform parameters for the three OB projection neurons subtypes include (***B***) injected current density needed to evoke an AP, (***C***) membrane potential at which the AP was evoked, (***D***) maximum voltage reached by the AP, (***E***) AP peak amplitude, (***F***) AP width at half the maximum height, (***G***) minimum voltage reached by the AP, and (***H***) peak amplitude of the AP AHP. Circles are individual cells, lines are mean ± SEM; *p < 0.05; **p < 0.01; ***p < 0.001; further quantification and statistical analysis in Table 1.

Next, we investigated AP threshold and waveform by injecting 10 ms of depolarizing current steps of increasing amplitudes (Fig. 4*A*). In line with findings from Burton and Urban (2014), the three cell types had similar AP waveforms, with only the peak amplitude being significantly smaller in eplTCs (Fig. 4*E*; Table 1). This difference is in line with similar maximum voltages reached but higher AP threshold in eplTC than in ML's cells (Fig. 4*B*–*D*; Table 1). Of note, when the AP threshold is normalized for capacitance, pMCs require smaller injected currents than pTCs to fire (Fig. 4*B*; Table 1). In contrast to the sodium channel-driven rapid depolarization and in line with molecular data (Zeppilli et al., 2021), the AP phases reliant on potassium conductance (width at half-height, WHH, after-hyperpolarization, AHP) do not differ between the three cell types, except for the higher minimum voltage reached by eplTCs (Fig. 4*F*–*H*).

**Table 1.** Intrinsic electrophysiological properties of OB projection neurons

Intrinsic electrophysiological properties							
	eplTC mean ± SEM, (*n*)	pTC mean ± SEM, (*n*)	pMC mean ± SEM, (*n*)	ANOVA/Kruskal– Wallis *p* value	Tukey's/Dunn's multiple comparisons *p* value		
					eplTC versus pTC	eplTC versus pMC	pTC versus pMC
Sag potentials properties							
Peak amplitude (mV)	71.97 ± 7.24 [10]	88.7 ± 15.82 [13]	67.27 ± 5.63 [28]	A, 0.24 (*F* = 1.46)	ns	ns	ns
Steady state (mV)	−121.3 ± 6.5 [10]	−109 ± 8.39 [13]	−115.5 ± 4.89 [28]	KW, 0.52	ns	ns	ns
Sag amplitude (mV)	11.20 ± 3.43 [10]	39.28 ± 17.80 [13]	11.53 ± 3.80 [28]	KW, 0.63	ns	ns	ns
Sag Index	0.86 ± 0.05 [10]	0.74 ± 0.09 [13]	0.86 ± 0.03 [28]	KW, 0.74	ns	ns	ns
APS properties							
Current threshold (pA/pF)	9.63 ± 1.64 [13]	5.46 ± 0.87 [13]	3.06 ± 0.37 [27]	KW, <0.001	ns	***	*
Voltage threshold (mV)	−30.22 ± 2.18 [13]	−37.26 ± 2.76 [13]	−43.14 ± 1.14 [27]	A, <0.001 (*F* = 13.14)	ns	***	ns
Maximum voltage (mV)	10.48 ± 2.01 [13]	14.89 ± 2.24 [13]	14.10 ± 1.76 [27]	KW, 0.40	ns	ns	ns
Peak amplitude (mV)	40.17 ± 1.83 [13]	58.56 ± 4.60 [13]	58.29 ± 2.06 [27]	KW, <0.001	**	***	ns
WHH (ms)	0.80 ± 0.08 [13]	0.93 ± 0.06 [13]	0.83 ± 0.03 [27]	KW, 0.25	ns	ns	ns
Minimum voltage (mV)	−46.22 ± 2.05 [15]	−54.03 ± 2.30 [11]	−56.26 ± 1.87 [27]	A, 0.003 (*F* = 6.41)	ns	**	ns
AHP on amplitude (mV)	17.13 ± 1.57[15]	12.61 ± 1.18 [11]	16.59 ± 1.10 [27]	A, 0.08 (*F* = 2.58)	ns	ns	ns
Repetitive firing properties							
Rheobase (pA/pF)	3.03 ± 0.69 [14]	1.59 ± 0.29 [11]	1.82 ± 0.31 [28]	A, 0.08 (*F* = 2.58)	ns	ns	ns
Slope I/O curve (Hz/pA/pF)	7.98 ± 1.38 [13]	6.33 ± 1.24 [11]	7.21 ± 1.01 [26]	A, 0.72 (*F* = 0.34)	ns	ns	ns
First AP delay (ms)	109.8 ± 20.6 [15]	169.5 ± 42.1 [11]	161.1 ± 29.1 [27]	KW, 0.78	ns	ns	ns
*n* APs at rheobase	8.14 ± 2.95 [14]	5.36 ± 1.22 [11]	8.64 ± 2.56 [28]	KW, 0.86	ns	ns	ns
Max number of APs	29.87 ± 4.76 [15]	22.27 ± 3.65 [11]	25.321 ± 2.67 [28]	KW, 0.67	ns	ns	ns
Max frequency (Hz)	110.9 ± 28.1 [15]	131.2 ± 25.4 [11]	80.1 ± 7.3 [28]	KW, 0.19	ns	ns	ns
ISI CV at max firing	0.19 ± 0.03 [14]	0.47 ± 0.16 [11]	0.19 ± 0.02 [27]	KW, 0.20	ns	ns	ns
ISI CV2 at max firing	0.10 ± 0.13 [14]	0.27 ± 0.10 [11]	0.11 ± 0.01 [27]	KW, 0.33	ns	ns	ns

Mean values ± SEM and number of cells (n) of sag currents, AP properties, and repetitive firing properties for eplTCs and ML's pTCs and pMC. Statistical differences between cell types were calculated independently with a one-way ANOVA with Tukey's post hoc correction for normally distributed data (A) or with a Kruskal–Wallis test with Dunn's post hoc correction for non-normally distributed data (KW). Individual data points and example traces are presented in Figures 3, 4, and 6.

**Figure 5.**
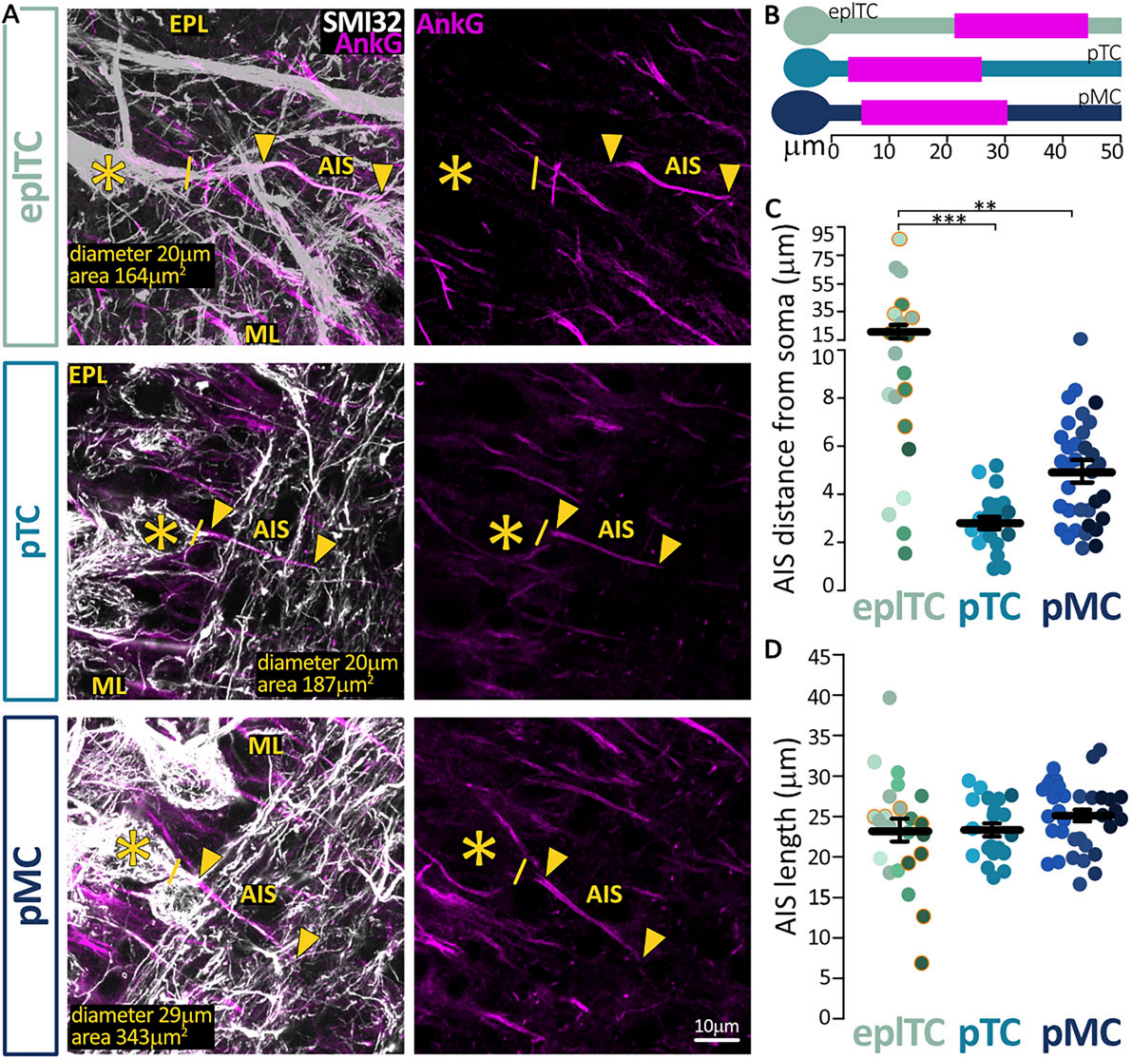
EPL cells have similarly long but more distal axon initial segments than ML neurons. ***A***, Example maximum intensity projection images of eplTC, pTC, and pMC neurons visualized via SMI-32 immunolabel (white) with an identified AnkG-positive AIS (magenta, arrows). The solid line indicates the emergence of the axonal process from the soma (asterisk). EPL, external plexiform layer; ML, mitral layer. ***B***, Mean AIS (magenta) start and end position for each group. ***C, D***, AIS distance from soma and length in eplTCs (green, *n* = 23); pTCs (cyan, *n* = 21); and pMCs (blue, *n* = 34). Circles represent individual cells, different color shades represent different mice; orange border indicates AIS originating from dendrite; lines are mean ± SEM; **p < 0.01; ***p < 0.001.

In summary, despite being more electronically compact (i.e., able to integrate electrical signals over a smaller spatial area or with fewer/shorter distinct components), eplTCs require more current to fire an AP which however has a similar shape to ML cell APs. To investigate whether this threshold difference may be due to morphological differences at the AP initiation site, the axon initial segment (AIS), we performed immunohistochemistry against the AIS master organizer and label AnkG (Fig. 5*A*; Kole et al., 2007; Bender and Trussell, 2012; Leterrier, 2018; Galliano et al., 2021).

We found no difference in AIS length between the three cell types (eplTC, 23.28 ± 1.41 µm; pTC, 23.28 ± 0.80 µm; pMC, 25.07 ± 0.72 µm; ANOVA nested on mouse *F_(_*_2,11)_ = 0.5529; *p* = 0.59; Fig. 5*B*,*D*). Conversely, eplTCs' AISes are extremely distal compared with those of principal neurons in the ML (eplTC, 21.19 ± 4.78 µm; pTC, 2.88 ± 0.25 µm; pMC, 5.02 ± 0.46 µm; log-transformed data for ANOVA nested on mouse, *F_(_*_2,11)_ = 14.16; *p* < 0.001; post hoc with FDR correction, eplTC vs pTC *p* < 0.001; eplTC vs pMC *p* = 0.003; pTC vs pMC *p* = 0.13; Fig. 5*C*). Recent work has identified axons originating from dendrites not just in inhibitory bulbar interneurons but also in pyramidal cells in the cortex (Galliano et al., 2021; Hodapp et al., 2022), and such dendritic origin can correlate with a more distal AIS location. However, we confirmed that while 8/23 eplTCs had dendritic axons, their AISes were not different from the AISes of the 15/23 eplTCs in terms of distance from soma, length, or diameter (orange shading in Fig. 5*C*–*D*, nested *t* tests; all *p* > 0.09). Importantly, the diameter of both the proximal axon and AIS were identical not just between eplTCs with somatic or dendritic-origin AISes, but among all cell types (ANOVA nested on mouse, *F_(_*_2,10)_ = 1.28; *p* = 0.32; *F_(_*_2,10)_ = 1.47; *p* = 0.28, respectively).

Literature suggests that, at comparable diameters and lengths (Goethals and Brette, 2020), excitability reduces the further the AIS is from the soma as more charge is required to overcome charge dissipation and generate an AP (Yamada and Kuba, 2016). Thus, this morphological data could at least partially explain the higher firing threshold in eplTC but fails to account for the difference in firing threshold recorded between pMCs and pTCs in the ML.

### Repetitive AP properties are comparable across putative cell types

**Figure 6.**
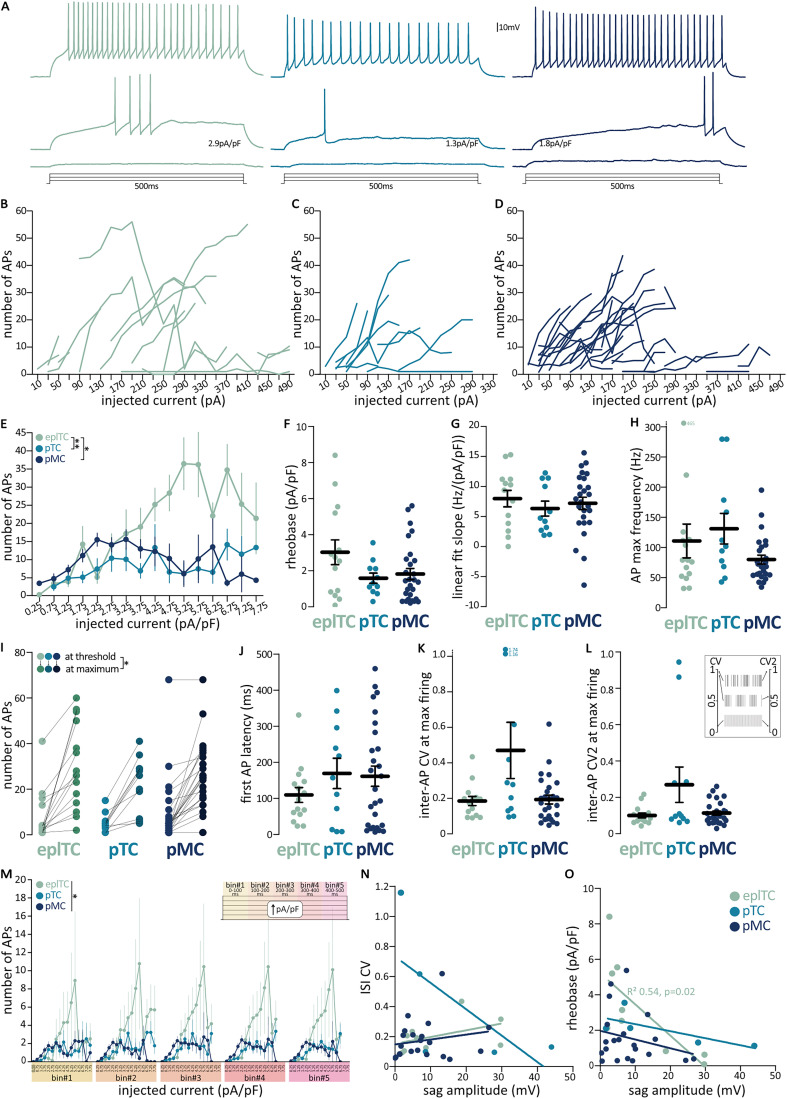
Comparable repetitive AP firing among OB projection neurons. ***A***, Example traces of the membrane voltage response to 500 ms depolarizing current injections at 300 pA, rheobase, and max AP firing in eplTCs (green, *n* = 15); pTCs (cyan, *n* = 11); and pMCs (blue, *n* = 27). ***B–D***, Raw input–output plots showing the number of APs fired at each current injection in individual neurons. ***E***, Mean number of APs and SEM at each current density (i.e., injected current normalized for cell capacitance) in eplTCs (green), pTCs (cyan), and pMCs (blue). Repetitive firing parameters include (***F***) rheobase, (***G***) slope of the number of APs versus current density input–output curves, (***H***) maximum AP firing frequency, (***I***) number of APs at threshold (light shades) and at maximum firing (dark shades), (***J***) latency of the first AP at the current injection level where the max AP number was fired, (***I, J***) CV of the ISIs (CV) and of adjacent ISIs (CV2; see inset for graphical description) at the current injection level where the max AP number was fired. ***M***, Input–output curve with the 500 ms current injection divided into five 100 ms bins (see inset). ***N–O***, Correlation of sag amplitude with firing properties. Circles and thin lines are individual cells; thick black lines are mean ± SEM; *p < 0.05; **p < 0.01; further quantification and statistical analysis in Table 1.

To investigate the rate and temporal coding in projection neurons, we injected longer current steps (500 ms) of increasing intensity (Fig. 6*A*–*D*). To account for different cell capacitance, we constructed input–output curves with injected current density as the independent variable (two-way ANOVA; effect of cell type *F_(_*_27-220) _= 27.08; *p* = 0.0015; effect of current density *F_(_*_14-220) _= 2.24; *p* = 0.07; effect of interaction *F_(_*_28-220) _= 2.06; *p* = 0.002; Tukey's multiple comparisons eplTC vs pTC *p* = 0.014; eplTC vs pMC *p* = 0.16; pTC vs pMC *p* = 0.52; Fig. 6*E*). From them, we extracted rheobase (Fig. 6*F*) and the rate of rise by fitting a line and calculating its slope (Fig. 6*G*). In line with the current and voltage threshold results discussed above, also with these longer-lasting injections, eplTC seemingly took longer than ML cells to fire APs but the rheobase values did not reach significance (Kruskal–Wallis test, *p* = 0.08), and all three cell types had similarly steep input–output relationships (Table 1). We also found no difference between the three cell types in the latency to fire the first AP, in the number of APs threshold or at maximum firing, nor in the maximum AP frequency (Fig. 6*H*–*J*; Table 1). Both in vivo and ex vivo preparations have shown that TCs fire more irregularly than MCs (Burton and Urban, 2014; Fourcaud-Trocmé et al., 2022). To investigate firing regularity, we calculated both the ISI CV (where a high ISI CV indicates irregular firing, Fig. 6*K*) and the CV of adjacent intervals (CV2, sensitive to regularity within a burst; Fig. 6*L*). Surprisingly, we found no differences between the three cell types in each measure, both at maximum firing (Table 1) and rheobase (data not shown). Moreover, we found that while eplTCs are more excitable, all three cell types fire similar number of APs at the beginning, middle, and end of the current injection (bin average AP number two-way ANOVA, effect of cell type *F*_(2,125) _= 3.58; *p* = 0.03; effect of current density *F_(_*_4,125) _= 0.08; *p* = 0.99; effect of interaction *F_(_*_8,125) _= 0.05; *p* = 1.00; Fig. 6*M*). Finally, we checked if the sag voltage amplitude correlated with either firing CV or rheobase (Burton and Urban, 2014) but found a significant correlation only for sag amplitude and rheobase in eplTCs (linear regression *R*^2 ^= 054; *F* = 8.22; *p* = 0.02; all other correlations *p* > 0.16; Fig. 6*O*). Taken together, our results indicate that when capacitance is accounted for, eplTCs have similar firing patterns to both pTCs and pMCs in the ML.

### Can putative cell type be accurately classified into discrete groups?

Despite the wealth of literature suggesting that TCs and MCs are physiologically different, our ex vivo data indicate that individual physiological passive and active properties are not sufficient to differentiate them. If individual properties cannot return a clean linear classifier, does the mitral versus tufted split appear when all these values are considered holistically?

**Figure 7.**
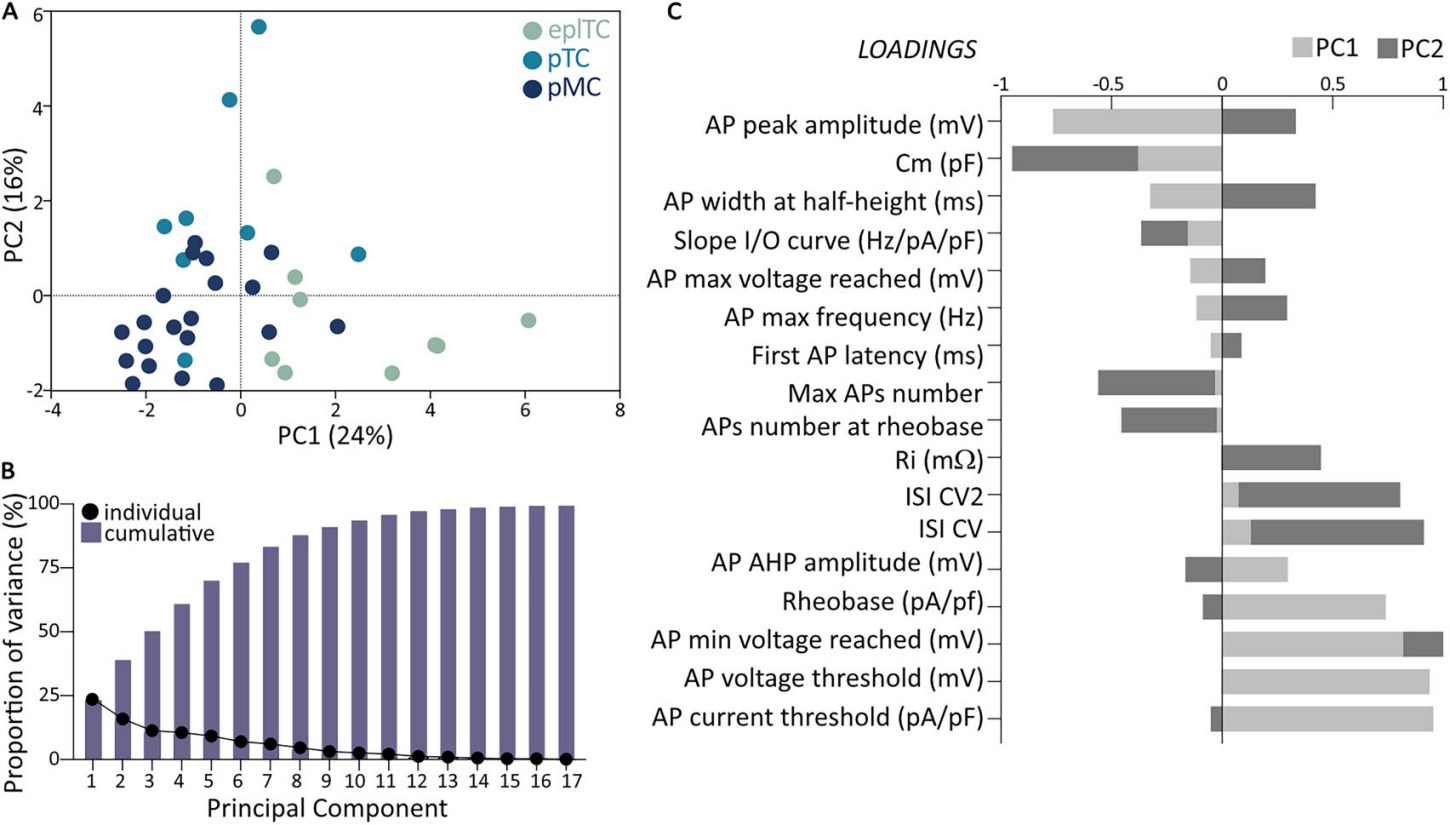
PCA of firing properties fails to reveal clear clustering of OB projection neurons. ***A***, PC score plot for eplTC (green, *n* = 9) and ML's pTC (cyan, *n* = 8) and pMC (blue, *n* = 20) based on passive properties and all measurements obtained from AP firing recordings (Figs. 2, 4, 6; Table 1). Each circle represents a cell plotted against its primary and secondary PC scores. ***B***, Individual (circles) and cumulative (bars) proportion of variance explained by each PC. ***C***, Loading scores for all variables showing their respective contributions to PC1 (light gray) and PC2 (dark gray). See also Extended Data Figure 7-1.

To answer this question and determine the source of the variation in our dataset, we performed a PCA including all measurements extracted from passive properties and AP firing protocols (Fig. 7*A*), after confirming that animal age did not produce distinct clusters (Extended Data Fig. 7-1). The first three PCs cumulatively accounted for 50% of the variance, and the most influential loading scores were connected to AP amplitude and threshold, as well as capacitance (Fig. 7*B*,*C*). While on average eplTCs and pMCs clustered at opposite ends of the PC1 axis and pTCs were more narrowly grouped around the middle, we found considerable overlap between the three cell types. In summary, the PCA failed to return clear clustering but suggested a more gradual continuum of heterogeneous properties in OB projection neurons.

10.1523/ENEURO.0407-24.2025.f7-1 Fig 7-1 **Animal age impact on PCA of firing properties.** As in figure 7A, principal component (PC) score plot for principal projection neurons based on passive properties and all measurements obtained from cells with stable passive properties and AP firing recordings (Figures 2-4-6, Table 1). Each circle represents a cell plotted against its primary and secondary PC scores and it has been colour-coded to indicate the animal age, ranging from P20 (grey) to P68 (purple). Download Fig 7-1, TIF file.

## Discussion

In this study we compared the morphological and functional properties of ex vivo murine bulbar principal neurons across deep EPL and ML using classification approaches based on often-used soma size threshold and unbiased clustering of membrane capacitance. We were unable to conclusively segregate pMCs and pTCs within the ML, but we confirmed earlier findings that very large ML cells are overall different from TCs in the EPL. Historically, smaller cells in the ML and cells with their soma only partially in the ML have been excluded from analysis because of their ambiguous identity (Burton and Urban, 2014; Nagayama et al., 2014). If these “transitional” cells are ignored, a very clear mitral/tufted split emerges. However, when they are included, such stark classification blurs as summarized by our PCA of AP properties which returned a polarized continuum rather than defined clusters. Taken all together, these data suggest that, besides the often-unavailable connectivity profile, anatomy paired with size and location is still the best classifier of bulbar principal neurons, which are overall extremely heterogeneous.

### Age, recording conditions, and analysis methods strongly influence AP firing

Both ex vivo and in vivo studies have shown that TC cells are more excitable than MCs and that they have a latency to fire in response to OSN inputs (Burton and Urban, 2014). While for 500-ms-long current injections we observed higher numbers of APs in eplTC input–output curves, our dataset did not replicate this higher excitability finding when looking more granularly at single AP thresholds. This discrepancy is likely due to both experimental and analysis differences. Indeed, contrary to Burton and Urban (2014), we recorded at lower physiologically relevant temperatures without synaptic blockers in postweaning mice and then normalized the injected shorter-lasting current injections for cell capacitance, which is by definition very different between the groups. Both age and synaptic blockers have been shown to be strong modulators of MC firing (Smithers et al., 2017; Van Hook, 2020; Tufo et al., 2022), which with increasing age becomes more attuned to high-frequency stimuli (Yu et al., 2015) and as such can easily explain the discrepancy. Moreover, our AIS morphological data are in line with the higher firing threshold in eplTCs. The AIS location, which together with its morphology has been shown to correlate best with somatic threshold than rheobase, was very distal in eplTCs. Given the similar proximal axon diameters in eplTCs and ML cells, this suggests that eplTCs do not operate in a high coupling regime with the soma and thus need more charge to initiate firing (Brette, 2013; Goethals and Brette, 2020). Furthermore, despite variations in recording conditions, our dataset mirrored the heterogeneity in sag potentials reported previously (Angelo and Margrie, 2011; Burton and Urban, 2014) and in general in most single AP and repetitive firing parameters. When comparing our results with in vivo studies, which primarily used rats of various ages as well as transgenic mice, we encountered several challenges. As expected, these studies are highly heterogeneous, with differences in methodology and classifications of MCs and TCs, and none report capacitance (Cang and Isaacson, 2003; Abraham et al., 2010; Phillips et al., 2012). Comparing firing frequency is not feasible due to methodological differences: we induced depolarizing current, while in vivo studies recorded spontaneous firing, which likely accounts for the higher frequencies observed in our study. For instance, Cang and Isaacson (2003) recorded a frequency of 2.8 ± 0.7 Hz in M/TCs, while Phillips et al. (2012) reported 57.8 ± 16.9 Hz. However, our input resistance for pMC (110 ± 13 MΩ) aligns with the value reported by Cang and Isaacson (115.0 ± 16.0 MΩ) in young wild-type rats. In summary, while our study is largely consistent with both ex vivo and in vivo work, the differences in experimental approaches, animal models, and ages presented challenges in making direct comparisons.

### Mitral, tufted, and everything in between: gray areas in the classification of bulbar principal neurons

The cell-to-cell variability within and between putative subclasses that we report here—where we intentionally avoided removing outliers to present the full range of recorded properties—stresses that bulbar principal neurons are a heterogeneous population (Zeppilli et al., 2021). Including both the analysis of the soma size in the fixed tissue and the firing properties, our dataset failed to return a clear separation between pMCs and pTCs in the ML, and even a ML versus deep EPL classification is somewhat blurred. This is not surprising because, while a traditional location + 20-µm-diameter classifier is appealing, there is accumulating evidence of overlap in the molecular and genetic profiles of MCs and TCs. For example, they heterogeneously express GABA_A_ receptors and voltage-gated potassium channels (Panzanelli et al., 2005), and while MCs and TCs can be classified at the level of transcriptomics, cell-type–specific modules of gene regulation fail to granularly class MCs and TCs (Zeppilli et al., 2021). Indeed, it is thought that their biophysical diversity is at least partly due not to genetic programs but to experience-dependent factors which have the potential to expand their heterogeneity (Padmanabhan and Urban, 2010; Angelo et al., 2012; Tripathy et al., 2013).

Importantly, OB principal neurons also differ in their morphology of lateral dendrites and their axonal projections and have consequent differences in synaptic connectivity (Christie et al., 2001; Nagayama, 2010; Gire et al., 2012; Nagayama et al., 2014; Geramita et al., 2016; Liu et al., 2019; Imamura et al., 2020; Jones et al., 2020). The combination of structural, functional, intrinsic, and synaptic properties likely returns not two clean groups—MCs and TC—but multiple subgroups (Padmanabhan and Urban, 2010; Angelo and Margrie, 2011; Kikuta et al., 2013; Goaillard and Marder, 2021; Zeppilli et al., 2021). This broad diversity could enable the OB to parallelly represent the wide odor and concentration space (Lee et al., 2023) via spatially and temporally distributed ensembles of active neurons (Uchida et al., 2014; Geramita et al., 2016; Shmuel et al., 2019).

### Heterogeneity as a key odor processing tool

For most sensory systems, the stimulus space is relatively well known (Huberman and Niell, 2011; King et al., 2015; O’Connor et al., 2021). Conversely, while we have long defined the number colors or sound frequencies mice—or humans—can perceive, the exact size of the olfactory stimulus space remains elusive. What we know is that, given the vast number of potentially detectable chemicals and their nonlinear combinations, the range of odors that animals can detect is truly large (Meister, 2015; Lee et al., 2023). This remarkable capacity is even more remarkable considering that the OSNs continuously regenerate throughout life (Schwob, 2002), a process that raises fascinating questions about how stable perception is maintained despite the constant turnover of the peripheral sensor. Olfactory processing is further unique because it eschews a thalamic relay and information only takes two synapses to go from the nose to cortex and other higher areas (Shepherd, 2004).

Given this stimulus and sensor complexity and such paired down relay anatomy, it is not surprising that the OB needs multiple parallel channels to process odors. It is thus tempting to speculate that heterogeneity of genes, morphologies, intrinsic properties, and synaptic connections is used throughout the olfactory system—principal neurons as well as OSNs and interneurons (Godfrey et al., 2004; Antal et al., 2006; Lledo et al., 2008; Galliano et al., 2018)—to sense, process, and classify olfactory information.

## Synthesis

Reviewing Editor: Arianna Maffei, Stony Brook University Decisions are customarily a result of the Reviewing Editor and the peer reviewers coming together and discussing their recommendations until a consensus is reached. When revisions are invited, a fact-based synthesis statement explaining their decision and outlining what is needed to prepare a revision will be listed below. The following reviewer(s) agreed to reveal their identity: Michele Dibattista.

There is strong agreement between the reviewers that the study tackles important questions in the field, experiments are carefully executed and the data quality is high.

An important concern regards the broad age range at which recordings were performed and the possibility that this may be a factor influencing variability in cell properties. It is important to clarify this issue as it influences the conclusions of the study.

Additional clarifications about specific parameters and analyses would help support the take home message. Text edits that streamline descriptions and provide a crisp and succinct presentation of findings and conclusions would increase the readability of the manuscript and its access to non-experts.

Specific point-by-point comments by the reviewers are included below.

Reviewer #1

The manuscript is interesting, experiments are carefully performed and presented, providing a thorough characterization of tufted and mitral cells in the mouse olfactory bulb. The main result, based on immunostainings and electrophysiology ex vivo, is that these cell 'types' are very diverse. Properties partially overlap, not allowing to easily differentiate cell types only based on electrophysiological properties, quantified with unsupervised algorithms. Authors deduce a large diversity of olfactory bulb cellular properties. The characterization of cellular diversity will be interesting in the future to ask how it plays a role in olfactory processing, and quantifications will be valuable to model the impact of diversity on network processing, as the authors advance in an interesting discussion that I also enjoyed reading where they discuss interesting functional implications of their findings.

I have a concern that authors should be able to address by analysis/maybe some rewriting as needed and minor revisions/ suggestions which I hope all will help improve the article.

1. My main concern, that would require some statistical testing/ maybe some rephrasing of the conclusions is the following: the range for the age of mice is very large, from juvenile to adults (21 to 68 days). Basically, if cellular properties show a large variability across developmental stages, which we actually expect from the literature on the bulb and other brain regions in these time windows, then it could as a result be very difficult to use these properties to predict a cell type, and claims on the magnitude of diversity may be somehow also including the diversity of properties throughout development.

Therefore, I think that authors should show that development is not a confusing factor, or if it is, which I would expect, to which degree, so that it appears legitimate to use parameters over those developmental times, that one actually expects to show a large developmental variation in this timeline (input resistance, capacitance, AP halfwidth etc.). How do authors approach this variability statistically and how do they rule out that it may be a major confusing factor in their analysis? Maybe difficulties to cluster cell types have a developmental contribution and clustering would be much better if more confined temporal window is used, and particularly, an adult one? The question is how much does it explain of the variability that authors interpret as diversity of cell properties.

A possibility would be doing a linear mixed effect model for the different parameters. Alternatively, given the large dataset, authors could compare variability in subsets of the dataset by 'chopping' it in smaller age groups. PCA may allow making a better prediction of cell type at a specific time point, maybe different across developmental stage. It would be also helpful if authors plot/ provide statistics for each cell type the dependence of parameters with age to visually evaluate trends, previous to performing additional statistical tests.

The article makes great recordings and stainings and will be, no matter the outcome, extremely valuable and I think should be published, but the interpretation/ of the paper may have to be adpated depending on the outcome of these analysis.

2. Whereas the article reads in general well, it is at times dense and long, maybe some parts can be included in the methods and not in the results section to ease reading.

3. How does the study compare to previous studies with data in vivo? Was there any in vivo attempt to do similar clustering as authors did here? If yes, how did it compare? I lack that olfactory-field specific background, and I may have missed this. It may be interesting putting in perspective more clearly.

4. p5 l4: First, what do the authors mean by holding voltage in current clamp? Second, after clarifying that they mean (the term is incorrect as it is), could authors justify this criterium that seems arbitrary?

5. Sometimes only some statistical comparisons are shown in figures. Are all combinations performed and corrected for multiple comparisons but only some are shown? These was not obvious looking at figures: do authors only show some comparisons or significant ones? it is probably somewhere and I may have missed it.

6. Given the importance of the capacitance as a classification criterium, and given the good quality capacitance measurements performed by authors, they may want to consider the following: different capacitive components, differentiated by their loading time-constants, may reveal the size of charged intrinsic compartments (dendrites, axons, see e.g. see Dieudonne 1998, Mejia-Gervacio et al. 2007) and additionally, also a third compartment corresponding to electrically-coupled compartments (Alcami and Marty, 2013). Authors may want to consider whether these may add information, and affect classification: adding the first, second and third capacitive components may provide electrical loading information on dendritic, axonal and coupled network structure, which could be different among cell types, and it may significantly improve classification/pinpoint the most 'predictive' capacitive component. Just a suggestion and idea, I do not consider authors have to do it if they do not wish to do it.

7. p6, l4: 'All PCAs were unsupervised'. Isn't PCA an unsupervised algorithm by definition?

Also, what is meant by unbiased k-means? Bias in the initialization or number of clusters evaluated by e.g. Silhouette?

8. I may have missed it, the resting membrane potential or resting firing rate are classical values used in many regions as a criterium to classify cells, which may be relevant to include if authors have the quantifications at hand. Otherwise also commenting on these parameters may be interesting.

9. It is great that authors perform recordings and systematically fill and reconstruct cells to check for morphological integrity. Were any cells excluded because of severe neurite cuts? Was there any quantitative criterium to assess morphological integrity? (e.g. cut axon less than x um from soma, number of dendrites ...)

10. I am unsure about the normalization by capacitance, could this normalization reduce prediction to a relevant parameter for classification? Also, a more fundamental question, should capacitance be the normalization parameter if any, instead of input conductance if we refer to evaluated parameters that are measured well beyond capacitive loading constants like firing rate?

11. Figure legends: I found missing panel letters in fig.6. Authors may want to check other figure legends.

11. Minor writing suggestions/typos

p3, l 34: 'of the brain in sensory systems' in an unclear phrasing

p4, l 24: that's a lot, was it 200kHz?

fig. 1.

- 22 or 24 data points?

- Are p values corrected for multiple comparisons in the figure?

- I am confused about the schematics: is that a conventional representation of a recording setup/amplifier? Shouldn't it be a triangle?

p7, l43: 'algorithm, which algorithm'

p9, l19: bar -- but?

Reviewer #2

The authors, through a series of elegant experiments, showed that projection neurons in the olfactory bulb possess biophysical properties that define them as an electrical continuum rather than distinct clusters. I found the work very interesting and it offers "food for thought" about OB circuitry.

I only have minor comments/questions:

Page 5, line 4: In current-clamp mode, experiments were only evaluated if their holding voltage was -60 {plus minus} 3mV.

Was this close to the different cell's resting membrane potential? Was the holding current for the different cells within the range described at page 4 line 28?

Figure 1: It would probably benefit from adding an explanatory image next to panel A or a panel outlining the different ROIs used to target the various cell types in different OB locations, from which the authors then measured the morphological parameters.

Figure 2, Panel C, please increase the dot size and make the marker for the centroids in bold or use a more visible marker.

Page 9, line 21: in summary, despite being more electronically compact,...

Please explain what electronically compact means

Figure 4 Panel G in the legend: should the minimum not the maximum as reposted in the legend.

The statistical analysis of the input-output curve shows that there is an effect of the cell type, which means that the output (number of APs) is different between the three cell types, is it correct?

Figure 6 and legend in the text are cut at panel L. While the Figure 6 and the legends after the references should be correct.

Page 14, line 12: we recorded at lower physiologically-relevant temperatures

In the methods the authors reported a recording temperature of 30 degree Celsius, is there a reason for choosing to record at 30 and not 37?

Page 15, from line 9: Conversely we still do not know how many odors a rodent - or human - could potentially detect, but we know that the range is truly vast (Meister, 2015; Lee et al., 2023) and that it does so via a regenerating peripheral sensor (Schwob, 2002).

Probably the sentence is too concise and needs further elaboration.

## Author Response

Manuscript eN-NWR-0407-24 - Response to reviewers Synthesis Statement for Author (Required):

There is strong agreement between the reviewers that the study tackles important questions in the field, experiments are carefully executed and the data quality is high. An important concern regards the broad age range at which recordings were performed and the possibility that this may be a factor influencing variability in cell properties. It is important to clarify this issue as it influences the conclusions of the study. Additional clarifications about specific parameters and analyses would help support the take home message. Text edits that streamline descriptions and provide a crisp and succinct presentation of findings and conclusions would increase the readability of the manuscript and its access to non-experts. Specific point-by-point comments by the reviewers are included below.

We thank the reviewers for their genuinely constructive feedback and for recognizing the importance of the study and the high quality of the data. We appreciate the concern regarding the broad age range at which recordings were performed and have clarified this aspect below after supplementary analysis. We have also provided additional clarifications on specific parameters and analyses to strengthen the support for our conclusions.

We agree that streamlining the text to present our findings and conclusions more succinctly will improve the manuscript's readability and accessibility. We have revised the manuscript, accordingly, considering the specific point-by-point comments provided by the reviewers.

We are confident that these changes - which included further analysis, graphical changes, and rewriting - have enhanced the clarity and quality of our manuscript, which we hope is now suitable for publication.

Reviewer #1 The manuscript is interesting, experiments are carefully performed and presented, providing a thorough characterization of tufted and mitral cells in the mouse olfactory bulb. The main result, based on immunostainings and electrophysiology ex vivo, is that these cell 'types' are very diverse. Properties partially overlap, not allowing to easily differentiate cell types only based on electrophysiological properties, quantified with unsupervised algorithms. Authors deduce a large diversity of olfactory bulb cellular properties. The characterization of cellular diversity will be interesting in the future to ask how it plays a role in olfactory processing, and quantifications will be valuable to model the impact of diversity on network processing, as the authors advance in an interesting discussion that I also enjoyed reading where they discuss interesting functional implications of their findings.

I have a concern that authors should be able to address by analysis/maybe some rewriting as needed and minor revisions/ suggestions which I hope all will help improve the article.

We thank the reviewer for their thoughtful and encouraging feedback. We are pleased to hear that the manuscript was found to be interesting and that the experiments were carefully performed and presented. We are also grateful for the positive comments regarding the discussion and agree that future investigations into this diversity will be essential for understanding its role in network processing.

1. My main concern, that would require some statistical testing/ maybe some rephrasing of the conclusions is the following: the range for the age of mice is very large, from juvenile to adults (21 to 68 days). Basically, if cellular properties show a large variability across developmental stages, which we actually expect from the literature on the bulb and other brain regions in these time windows, then it could as a result be very difficult to use these properties to predict a cell type, and claims on the magnitude of diversity may be somehow also including the diversity of properties throughout development.

Therefore, I think that authors should show that development is not a confusing factor, or if it is, which I would expect, to which degree, so that it appears legitimate to use parameters over those developmental times, that one actually expects to show a large developmental variation in this timeline (input resistance, capacitance, AP halfwidth etc.). How do authors approach this variability statistically and how do they rule out that it may be a major confusing factor in their analysis? Maybe difficulties to cluster cell types have a developmental contribution and clustering would be much better if more confined temporal window is used, and particularly, an adult one? The question is how much does it explain of the variability that authors interpret as diversity of cell properties.

A possibility would be doing a linear mixed effect model for the different parameters. Alternatively, given the large dataset, authors could compare variability in subsets of the dataset by 'chopping' it in smaller age groups. PCA may allow making a better prediction of cell type at a specific time point, maybe different across developmental stage. It would be also helpful if authors plot/ provide statistics for each cell type the dependence of parameters with age to visually evaluate trends, previous to performing additional statistical tests.

The article makes great recordings and stainings and will be, no matter the outcome, extremely valuable and I think should be published, but the interpretation/ of the paper may have to be adpated depending on the outcome of these analysis.

We thank the reviewer for their insightful comments and suggestions. It is refreshing and encouraging to receive such constructive feedback.

We completely agree with the importance of developmental stages and life stages in contributing to cellular properties. To address this, we ensured that almost all our recordings were made in animals of consistent juvenile ages. Specifically, as you can see below in the figure on the left, most recordings were performed between P20 and P40. A small subset of cells (7 out of 92, or ~7%) was recorded from slightly older animals between P41 and P68, comprising 1 eplTC (green) and 6 pMCs (dark blue). Notably, the pMCs are the most abundant group in our dataset (42 cells), so even these older cells represent a minority within that group. This deviation occurred due to limitations during the COVID pandemic, which restricted animal availability, and we prioritized minimizing waste.

Prompted by your comments, we have conducted a re-analysis to compare these few cells from older animals with the vast majority of cells recorded from juvenile animals. As you can see with the age-coded PCA below on the right, we confirm that the seven cells from older animals are not clustered together but interspersed with the others. We have now included the exact proportions of cells recorded at each age in the revised manuscript on page 3.

We apologize for not including these details in the original submission and thank you again for highlighting this important aspect.

2. Whereas the article reads in general well, it is at times dense and long, maybe some parts can be included in the methods and not in the results section to ease reading.

We have taken this comment onboard and edited the manuscript accordingly by moving multiple paragraphs from the results to the methods (pages 3-6).

3. How does the study compare to previous studies with data in vivo? Was there any in vivo attempt to do similar clustering as authors did here? If yes, how did it compare? I lack that olfactory-field specific background, and I may have missed this. It may be interesting putting in perspective more clearly.

Our study used mice aged P20-P68, classifying mitral and internal tufted cells based on capacitance, with tufted cells in the lower 50% of the external plexiform layer (EPL). In contrast, most patching in vivo studies primarily used rats aged between P12 and 18 weeks (Cang &Isaacson, 2003; Angelo &Margrie, 2011; Phillips et al., 2012), or juvenile to adult mice (P40-P70; Abraham et al., 2010). These studies varied in their classification approaches, with some providing detailed classifications based on cell location and morphology, such as Phillips et al. (2012) and Cang &Isaacson (2003), while others, like Abraham et al. (2010), focused on mitral cells without distinguishing between cell types and did not report capacitance. In terms of input resistance, the value reported by Cang &Isaacson (2003) (115.0 {plus minus} 16.0 MΩ) in young wild-type rats is similar to that observed in our study (110 {plus minus} 13 MΩ) for mitral cells. However, Abraham et al. (2010) reported a much lower input resistance (32.9 {plus minus} 11.6 MΩ), which may be attributed to their use of a transgenic mouse model. Another parameter that we recorded and some of these studies reported is action potential frequency. The maximum frequency observed in our study for mitral cells (80 Hz) is higher than those reported in vivo. Cang &Isaacson (2003) recorded a frequency of 2.8 {plus minus} 0.7 Hz in mitral/tufted cells, while Phillips et al. (2012) reported a frequency of 57.8 {plus minus} 16.9 Hz. A direct comparison is difficult because our study used current injection to induce action potentials, while in vivo studies measured spontaneous activity, which is influenced by the physiological context and network interactions in the living brain.

In summary, while our study is largely in line with previous in vivo work, there are notable differences in experimental approaches, animals, and ages. This variation likely accounts for the differences in values observed. Importantly, our findings are not completely at odds with the in vivo literature. We have added additional sentences in the discussion to contextualize these comparisons, on page 10.

4. p5 l4: First, what do the authors mean by holding voltage in current clamp? Second, after clarifying that they mean (the term is incorrect as it is), could authors justify this criterium that seems arbitrary? Thank you for your comment. By 'holding voltage in current clamp,' we meant that we inject enough current to maintain the membrane potential at approximately -60{plus minus}3 mV during current-clamp conditions. We recognise that this terminology was a poor choice of words and have adjusted the text accordingly for clarity. The rationale for this approach was to standardise and hyperpolarise the cells to ensure that we were not starting with channels open, providing a consistent baseline across cells. This methodology is based on our previous published work, where the use of this holding potential helped minimise variability in the initial conditions, ensuring more reliable assessment of the cells' responses.

5. Sometimes only some statistical comparisons are shown in figures. Are all combinations performed and corrected for multiple comparisons but only some are shown? These was not obvious looking at figures: do authors only show some comparisons or significant ones? it is probably somewhere and I may have missed it.

We apologise for the confusion. In the figures, we have marked only the significant comparisons with a star, and all p-values and statistical tests, including post hoc comparisons, are reported in table 1. We have conducted all necessary comparisons and applied the appropriate corrections for multiple comparisons for all measurements. We have clarified this in the Methods section on page 6.

6. Given the importance of the capacitance as a classification criterium, and given the good quality capacitance measurements performed by authors, they may want to consider the following: different capacitive components, differentiated by their loading time-constants, may reveal the size of charged intrinsic compartments (dendrites, axons, see e.g. see Dieudonne 1998, Mejia-Gervacio et al. 2007) and additionally, also a third compartment corresponding to electrically-coupled compartments (Alcami and Marty, 2013). Authors may want to consider whether these may add information, and affect classification: adding the first, second and third capacitive components may provide electrical loading information on dendritic, axonal and coupled network structure, which could be different among cell types, and it may significantly improve classification/pinpoint the most 'predictive' capacitive component. Just a suggestion and idea, I do not consider authors have to do it if they do not wish to do it.

Thank you for the insightful suggestion. We agree that analysing different capacitive components and their time constants, as highlighted in the listed studies, could offer valuable insights into dendritic, axonal, and coupled network structures. However, implementing such an analysis would require specific recording protocols designed to isolate these components, such as those used by Alcami and Marty (2013), which included hyperpolarizing voltage steps in controlled voltage ranges and/or Ih pharmacology to minimize overlap with active currents. As our study did not employ these specific protocols, this type of detailed capacitive analysis is not feasible with our current dataset. For now, we have prioritized soma-level basic capacitance as a more straightforward and accessible measure for classifying cell types, something that most patch-clamp labs can directly obtain from typical recordings. Nonetheless, we appreciate the value of this approach and will consider incorporating it into future follow-up studies.

7. p6, l4: 'All PCAs were unsupervised'. Isn't PCA an unsupervised algorithm by definition? Also, what is meant by unbiased k-means? Bias in the initialization or number of clusters evaluated by e.g. Silhouette? You are of course right that PCA is inherently an unsupervised algorithm. The text has been updated to clarify that cell subtypes were assigned in a post-hoc manner after performing the PCA. As for k-means clustering, it is unbiased in terms of the number of clusters, with the optimal number determined using the silhouette coefficient. This clarification has also been added to the text.

8. I may have missed it, the resting membrane potential or resting firing rate are classical values used in many regions as a criterium to classify cells, which may be relevant to include if authors have the quantifications at hand. Otherwise also commenting on these parameters may be interesting.

We agree with the reviewer that resting membrane potential is indeed a commonly used parameter for classifying cells, but there are challenges in obtaining accurate measurements in whole-cell patch-clamp recordings. In previous studies, we have used the "quick and dirty" approach of measuring Vm immediately upon breaking into the cell at I=0, especially when comparing the same cell types that had undergone different manipulations. However, given that mitral and tufted cells differ in size, as is the premise of this study, measuring Vm in this way can be problematic. The rapid dialysis of the intracellular solution in large vs small sized somas can significantly affect the reading, leading to differential inaccuracies based on the varying rate of dialysis in cells of different sizes. For more precise assessment of Vm, perforated patch-clamp recordings be preferable, as these methods help preserve the intracellular environment and reduce artifacts from intracellular solution exchange. We opted to exclude this parameter to maintain the robustness and comparability of our dataset.

9. It is great that authors perform recordings and systematically fill and reconstruct cells to check for morphological integrity. Were any cells excluded because of severe neurite cuts? Was there any quantitative criterium to assess morphological integrity? (e.g. cut axon less than x um from soma, number of dendrites ...) Thank you for your comment. We did attempt to reconstruct some of the cells, as shown in Figure 1, but ultimately opted to maximise the use of slices by patching multiple cells in each slice. To ensure the morphological integrity of the cells, we added Alexa 488 to the intracellular solution. This allowed us to properly measure soma size and confirm that neurites were not cut under the field of view of the camera and microscope. The field of view for all the images taken on the previous setup was 100.90 µm x 179.43 µm, with a total area of 18,109.87 µm². While we cannot rule out that very apical tufts or lateral dendrites may have been cut, we believe that the portions near the recording pipette - those that contribute most significantly to the capacitance measurements that we took - were intact. We have clarified this point in the methods section, including the size of the field of view on page 5.

10. I am unsure about the normalization by capacitance, could this normalization reduce prediction to a relevant parameter for classification? Also, a more fundamental question, should capacitance be the normalization parameter if any, instead of input conductance if we refer to evaluated parameters that are measured well beyond capacitive loading constants like firing rate? Thank you for raising this point. We believe that normalizing by capacitance is necessary because the current density will differ significantly between pMC and pTC cells at the same injection step, making direct comparisons of input/output curves uninformative. By normalizing capacitance, we ensure that the data are comparable across cell types. However, we understand the importance of providing raw data for clarity, and we have included this in Figure 6B-D. The normalized averages and SEM, where we compare the three groups, are presented in Figure 6E. We hope this additional context helps address your concern.

11. Figure legends: I found missing panel letters in fig.6. Authors may want to check other figure legends.

We apologise to the reviewers for this, it was a typesetting mistake that arose from trying to embed the figures with legends in the main text for their ease of reading. We have now split figures from legends, which can be found fully copy edited at the end of the manuscript.

11. Minor writing suggestions/typos p3, l 34: 'of the brain in sensory systems' in an unclear phrasing Edited p4, l 24: that's a lot, was it 200kHz? Yes, 200 kHz is very high, but it ensures precise capture of action potential kinetics. File sizes remain small due to the short recording durations. fig. 1.

- 22 or 24 data points? We thank the reviewer for pointing this out! The correct value is in fact 23, the 24th data point was mistakenly labelled in dark blue in the figure, and 22 in the legend was clearly a typo. We have thoroughly reviewed all primary data and confirmed that the analysis and stats was performed correctly with 23 values. The figure legend has been corrected accordingly and the figure fixed.

- Are p values corrected for multiple comparisons in the figure? Yes, see table and response to point 5.

- I am confused about the schematics: is that a conventional representation of a recording setup/amplifier? Shouldn't it be a triangle? We apologise for this, we inadvertently copied from a previous paper's figure the stimulating electrode instead of the recording electrode. It is now a proper triangle. p7, l43: 'algorithm, which algorithm' Edited p9, l19: bar -- but? It was bar. Changed to "except for" to make it clearer.

Reviewer #2 The authors, through a series of elegant experiments, showed that projection neurons in the olfactory bulb possess biophysical properties that define them as an electrical continuum rather than distinct clusters. I found the work very interesting and it offers "food for thought" about OB circuitry.

We thank the reviewer for their kind words and positive feedback. We are pleased to hear that the work was found interesting and thought-provoking, particularly in its contribution to understanding olfactory bulb circuitry.

I only have minor comments/questions:

Page 5, line 4: In current-clamp mode, experiments were only evaluated if their holding voltage was -60 {plus minus} 3mV.

Was this close to the different cell's resting membrane potential? Was the holding current for the different cells within the range described at page 4 line 28? The holding voltage of -60 {plus minus} 3 mV was chosen based on the range of resting membrane potentials reported in the literature, both in vivo and ex vivo, particularly for mitral cells. We aimed to stay on the lower end of this range to minimize the risk of having already open conductances before any current injection. Resting membrane potential values for mitral cells reported in the literature include those from Burton &Urban (2014) at −53.9 {plus minus} 4.0 mV, Cang &Isaacson (2003) at −56.0 {plus minus} 1.2 mV, Phillips et al. (2012) at −51.3 {plus minus} 4.9 mV, Bischofberger &Jonas (1997) at −63.5 {plus minus} 0.6 mV, Pandipati &Schoppa (2018) at −53.8 {plus minus} 1.2 mV, Desmaisons et al. (1999) at −62.7 {plus minus} 1.7 mV, Zibman et al. (2011) at −53.4 {plus minus} 0.2 mV, and Palouzier-Paulignan et al. (2002) at −49 {plus minus} 8.36 mV, among others. As noted above, we required a stable starting point across cells to ensure reliable comparisons of firing thresholds. By using a holding voltage of -60 {plus minus} 3 mV, we established a consistent baseline, which minimized variability and facilitated more accurate comparisons of firing properties across the different cell types.

Figure 1: It would probably benefit from adding an explanatory image next to panel A or a panel outlining the different ROIs used to target the various cell types in different OB locations, from which the authors then measured the morphological parameters.

We have added ROIs outlines in dashed magenta on the representative cells for eplTC, pTC, and pMC in Figure 1 panel A.

Figure 2, Panel C, please increase the dot size and make the marker for the centroids in bold or use a more visible marker.

Done.

Page 9, line 21: in summary, despite being more electronically compact,...

Please explain what electronically compact means Added to the text: despite being more electronically compact (i.e., able to integrate electrical signals over a smaller spatial area or with fewer/shorter distinct components).

Figure 4 Panel G in the legend: should the minimum not the maximum as reposted in the legend.

Yes, thank you for spotting this. Now corrected in the figure.

The statistical analysis of the input-output curve shows that there is an effect of the cell type, which means that the output (number of APs) is different between the three cell types, is it correct? We thank the reviewer for pointing this out, we had indeed omitted the p values for the multiple comparisons in the input/output curves. These are now included in the text on page 9, and in the figure, significance is indicated with relevant stars.

Figure 6 and legend in the text are cut at panel L. While the Figure 6 and the legends after the references should be correct.

We apologise to the reviewers for this, it was a typesetting mistake that arose from trying to embed the figures with legends in the main text for their ease of reading. We have now split figures from legends, which can be found fully copy edited at the end of the manuscript.

Page 14, line 12: we recorded at lower physiologically-relevant temperatures. In the methods the authors reported a recording temperature of 30 degree Celsius, is there a reason for choosing to record at 30 and not 37? We chose to record at 30{degree sign}C rather than 37{degree sign}C due to technical constraints. At 37{degree sign}C, the slices deteriorate rapidly in our hands, likely due to the thinness of the slices, which caused them to overheat under the microscope. Still, we typically record at slightly higher temperatures, around 34{degree sign}C. However, this project was initiated during the pandemic, and we had to work without central heating due to ventilation concerns. The room was heated with electric heaters, and we could only achieve a temperature range of 30-32{degree sign}C due to the high ceiling and limited heating capacity. Importantly, this temperature range is consistent with many studies in the literature (e.g., including Palouzier-Paulignan et al. (2002) who recorded at 32{degree sign}C, Pandipati &Schoppa (2018) who used 31-33{degree sign}C, Pressler et al. (2007) at 30{degree sign}C, Isaacson &Strowbridge (1998) who used either room temperature (22-25{degree sign}C) or 30{degree sign}C) where a range from room temperature to 34-36{degree sign}C is commonly used.

Page 15, from line 9: Conversely we still do not know how many odors a rodent - or human - could potentially detect, but we know that the range is truly vast (Meister, 2015; Lee et al., 2023) and that it does so via a regenerating peripheral sensor (Schwob, 2002). Probably the sentence is too concise and needs further elaboration.

Edited and extended for clarity, now on page 11.
