# Supplementary figures and images for: Ex Vivo Functional Characterization of Mouse Olfactory Bulb Projection Neurons Reveals a Heterogeneous Continuum
Source: eNeuro. 2025 Feb 28;12(3):ENEURO.0407-24.2025. doi: 10.1523/ENEURO.0407-24.2025 (PMC11881907; doi:10.1523/ENEURO.0407-24.2025)

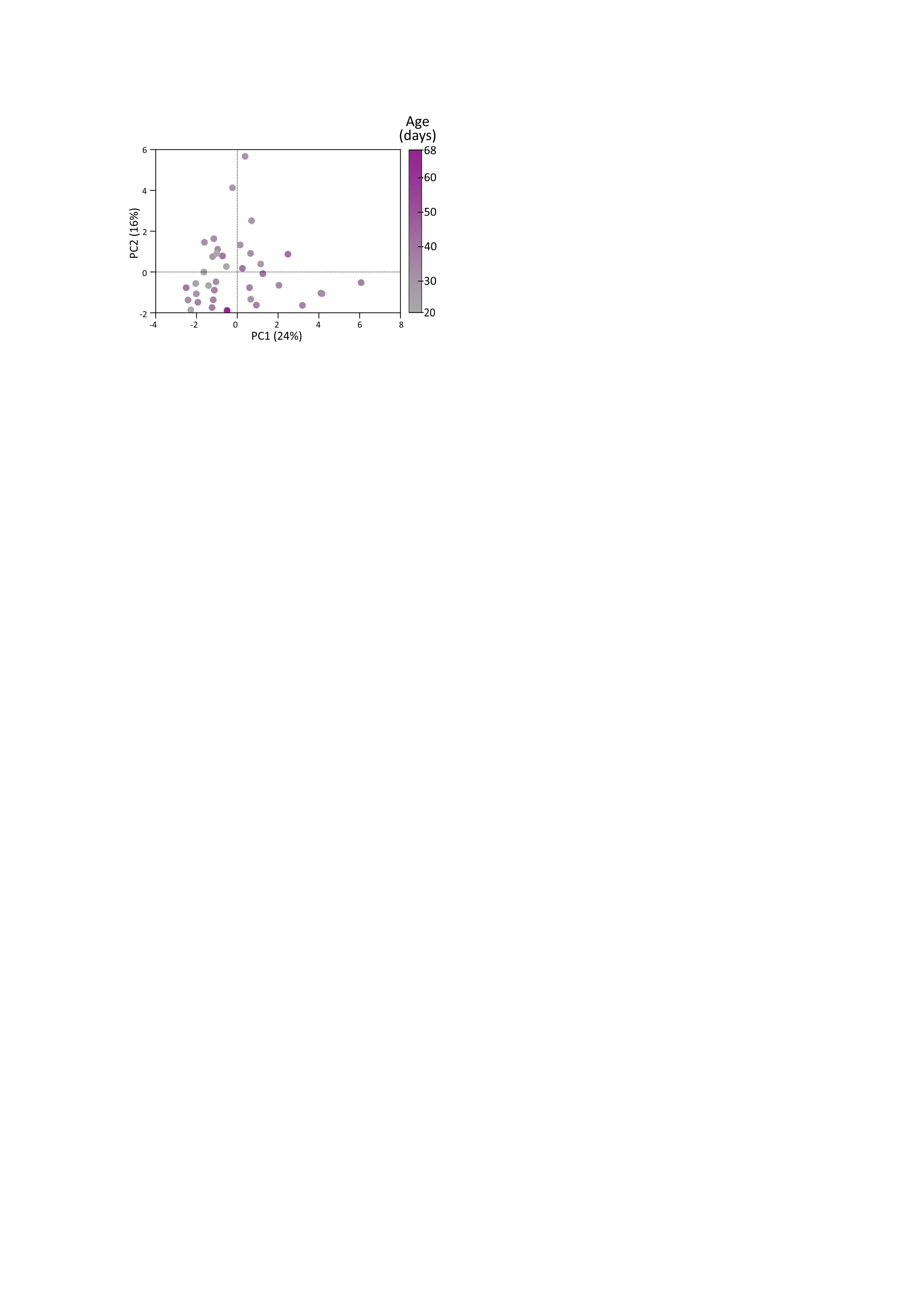

Supplement: Fig 7-1 — Animal age impact on PCA of firing properties. As in figure 7A, principal component (PC) score plot for principal projection neurons based on passive properties and all measurements obtained from cells with stable passive properties and AP firing recordings (Figures 2-4-6, Table 1). Each circle represents a cell plotted against its primary and secondary PC scores and it has been colour-coded to indicate the animal age, ranging from P20 (grey) to P68 (purple). Download Fig 7-1, TIF file. [file eneuro-12-ENEURO.0407-24.2025-s001.tif]
